# 1,3-Diketone Calix[4]arene Derivatives—A New Type of Versatile Ligands for Metal Complexes and Nanoparticles

**DOI:** 10.3390/molecules26051214

**Published:** 2021-02-24

**Authors:** Sergey N. Podyachev, Rustem R. Zairov, Asiya R. Mustafina

**Affiliations:** Arbuzov Institute of Organic and Physical Chemistry, FRC Kazan Scientific Center of RAS, 8 Arbuzov str., Kazan 420088, Russia; rustem02@yandex.ru (R.R.Z.); asiyamust@mail.ru (A.R.M.)

**Keywords:** calixarenes, 1,3-diketone, macrocycles, ligands, lanthanides, complexation, supramolecular chemistry, luminescence, nanoparticle

## Abstract

The present review is aimed at highlighting outlooks for cyclophanic 1,3-diketones as a new type of versatile ligands and building blocks of the nanomaterial for sensing and bioimaging. Thus, the main synthetic routes for achieving the structural diversity of cyclophanic 1,3-diketones are discussed. The structural diversity is demonstrated by variation of both cyclophanic backbones (calix[4]arene, calix[4]resorcinarene and thiacalix[4]arene) and embedding of different substituents onto lower or upper macrocyclic rims. The structural features of the cyclophanic 1,3-diketones are correlated with their ability to form lanthanide complexes exhibiting both lanthanide-centered luminescence and magnetic relaxivity parameters convenient for contrast effect in magnetic resonance imaging (MRI). The revealed structure–property relationships and the applicability of facile one-pot transformation of the complexes to hydrophilic nanoparticles demonstrates the advantages of 1,3-diketone calix[4]arene ligands and their complexes in developing of nanomaterials for sensing and bioimaging.

## 1. Introduction

Calix[n]arene chemistry had been a top of current interest from late 1980 to early 2000 [1,2,3,4,5,6], although up to now calix[n]arene backbones are still relevant as building blocks of different functional materials [7,8,9,10,11]. Calix[4]arenes, thiacalix[4]arenes and calix[4]resorcinarenes are the most widely applied scaffolds among other calix[n]arenes for embedding of different groups [12,13,14,15]. The stability of a *cone*-conformation, in turn, opens new opportunities for the preorganization of functional groups, which is of great advantage in developing of ligands and extractants [16,17,18]. The 1,3-diketone derivatives have been well-documented as both promising precursors in organic synthesis [19,20] and as promising compounds in drug design [21,22], although the greatest attention has been focused on their complex ability [23]. The latter is greatly affected by the structure of 1,3-diketones, since their complexation with metal ions results from the alkaline-driven diketone-to-diketonate transformation undergoing through the keto-enol equilibrium shift.

The complex ability of the 1,3-diketone-based ligands can be controlled by their structural diversifications based on both variation of the substituents and joining several 1,3-diketone moieties in one molecule leading to the so-called poly-diketones [24,25]. The latter have been documented as promising building blocks in development of the MOF-based structures [26,27].

Among other metal complexes, lanthanide 1,3-diketone derivatives have attracted the greatest interest due to the documented impact of the 1,3-diketonate ligands on sensitizing of lanthanide-centered luminescence through a ligand-to-metal energy transfer [23,28,29,30]. The aforesaid capacity prompts great diversity of 1,3-diketone derivatives as the ligands for lanthanide ions. The lanthanide complexes with 1,3-diketonates provide good basis for developing of sensors [31]. The sensing ability of lanthanide complexes with 1,3-diketonates has recently been exemplified by sensitivity of lanthanide-centered luminescence to temperature and pH changes, sensing of antibiotics, pressure-induced phase transition [32,33,34,35,36,37,38,39,40,41].

(1) Thus, the present review is focused on synthetic and structural aspects of calix[4]arene, thiacalix[4]arene and calix[4]resorcinarenes as scaffolds for embedding of 1,3-diketone moieties.
(2) The structural features of the cyclophanic 1,3-diketones will be correlated with their ability to complex lanthanide ions and to feed lanthanide-centered excited states through the ligand-to-metal energy transfer.
(3) The different modes of conversion of lanthanide 1,3-diketonate complexes from molecular to water-dispersible nanoparticulate forms will be also discussed herein as a prerequisite for their applicability for bio-sensing and bio-imaging. The structure–property relationships of the complexes in nanoparticulate forms will be correlated with their sensing and imaging functions.

## 2. Synthesis of the 1,3-Diketone Calix[4]arene Derivatives

**Scheme 1:**
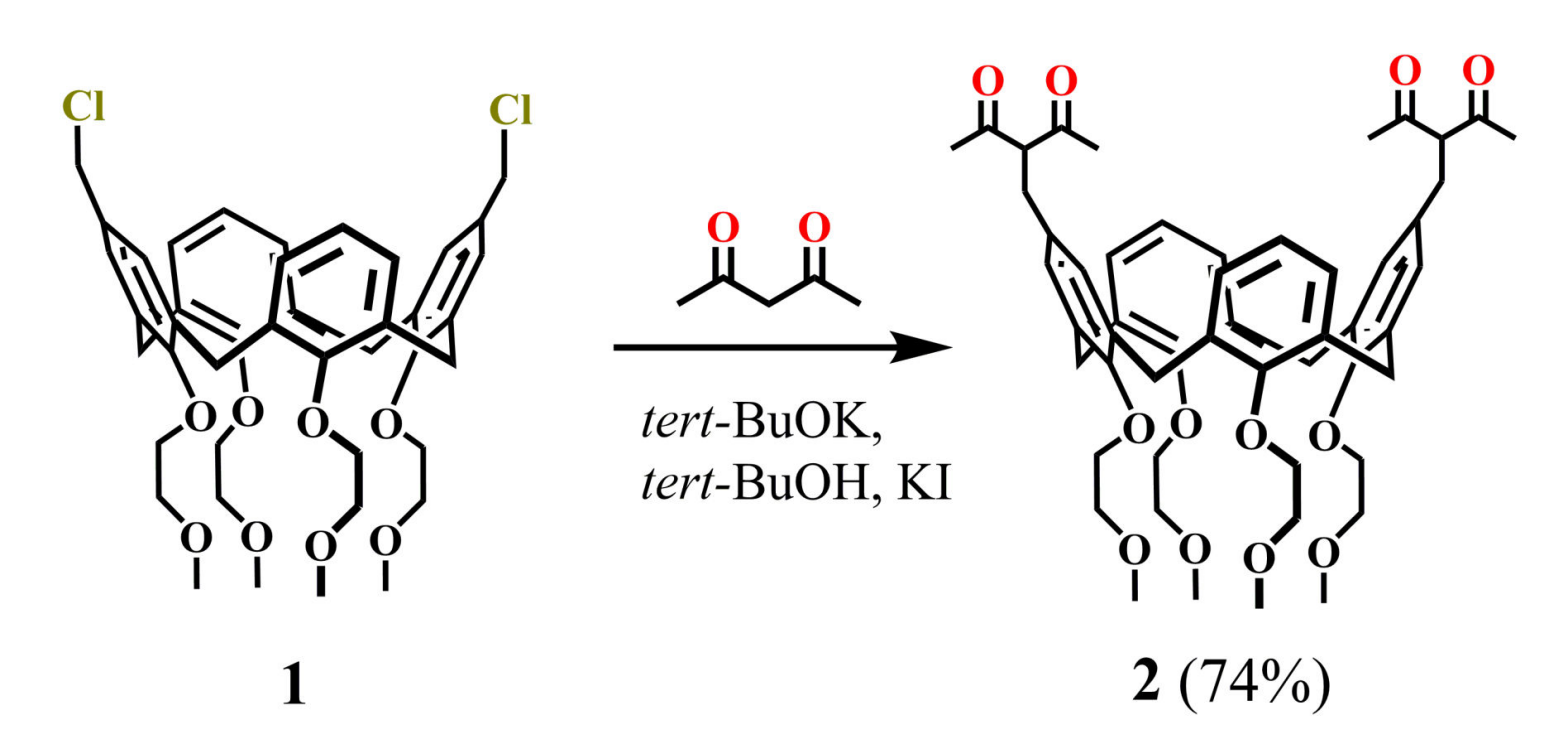
Preparation of bis-1,3-diketone calix[4]arene **2.**

Synthesis of the calix[4]arenes functionalized by 1,3-diketone groups was first reported by Shinkai group (1994) [42]. The addition of potassium *tert*-butoxide and KI to the dichloromethylated calix[4]arene **1** and acetylacetone (HAA) in *tert*-butanol solution (Scheme 1) resulted in the isolation of calix[4]arene **2** in a good yield (74%). Unfortunately, neither synthetic protocol nor spectral characteristics of the bis-1,3-diketone **2** were reported in the article apart from the scheme of the synthesis.

**Scheme 2:**
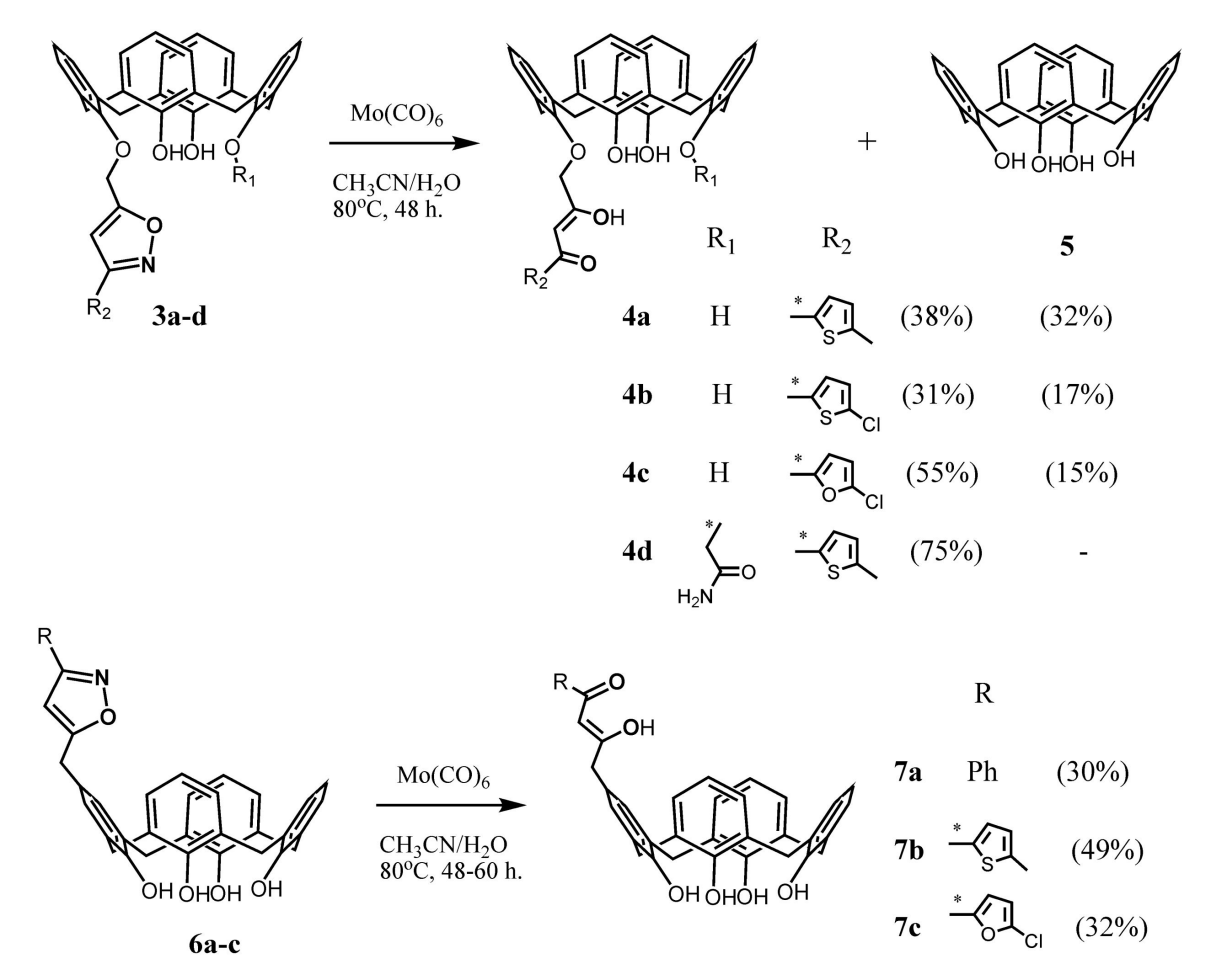
Preparation of mono-1,3-diketone derivatives of calix[4]arene **4a**–**d** and **7a**–**c**.

The synthetic strategy for the embedding of one 1,3-diketone group onto the upper or lower rims of calix[4]arenes was developed by Senthilvelan et al. (2006) a decade later [43] (Scheme 2). To obtain these compounds, the isoxazoline derivatives **3a**–**d** or **6a**–**c** were heated in acetonitrile in the presence of catalyst Mo(CO)_6_ and few drops of water, which was resulted in the ring opening and formation of 1,3-diketone derivatives **4a**–**d** and **7a**–**c**. The authors noted that along with the formation of the target products **4a**–**c**, the disproportionation of the initial isoxazoline derivatives also took place with a formation of a noticeable amount (15–32%) of unsubstituted calix[4]arene **5**. The yield of the target products **4a**–**c** and **7a**–**c** was only 30–55%. In the case of the compound **4d** bearing an amide group at the lower rim, the disproportionation did not occur, and the yield of the product reached 75%. The formation of the desired 1,3-diketones was not observed even under the use of the H_2_/Pd–C system to open the isoxazole ring.

This method, according to the authors opinion, is rather effective and convenient for the preparation of the calix[4]arenes bearing 1,3-diketone fragments both at the lower and upper rims of the macrocycle. However, it is worth noting a relatively low yield of the reaction, strong requirements for the choice of the catalyst, a rather effortful synthesis of the starting isoxazoline derivatives and a possibility of disproportionation of the starting reagents during the reaction as the factors complicating the usage of this approach for the synthesis of calix[4]arene 1,3-diketones.

**Scheme 3:**
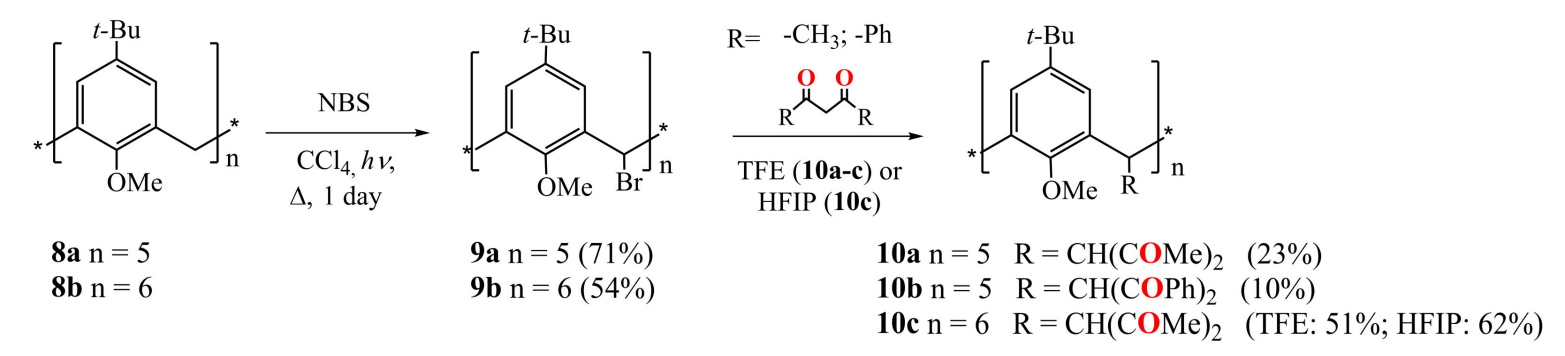
Preparation of penta-1,3-diketone derivatives of calix[5]arene **10a**,**b** and hexa-1,3-diketone calix[6]arene **10c**.

**Scheme 4:**
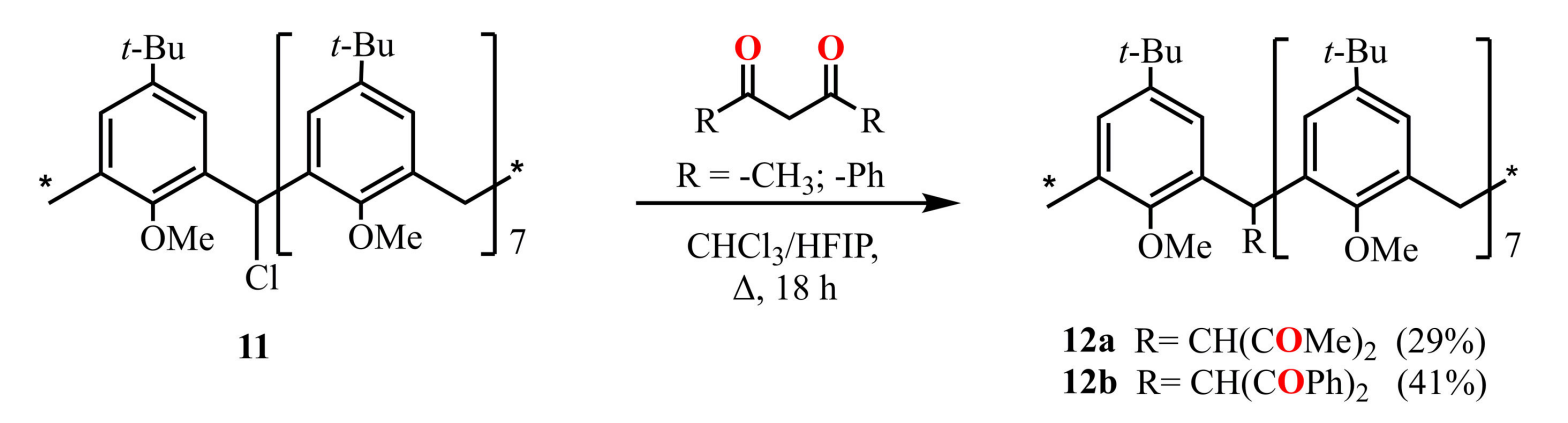
Preparation of mono-1,3-diketone derivatives of calix[8]arene **12a**,**b**.

Biali et al. were the first who functionalized the calix[4]arene (2011) [44], calix[5]arene (2009) [45], calix[6]arene (2008) [46] and calix[8]arene (2011) [47] by 1,3-diketone fragments at the bridging carbon atoms of the macrocycles (Scheme 3 and Scheme 4). At the first stage, one of two bridging hydrogen atoms in the starting methoxy-substituted calix[n]arenes **8a**,**b** was replaced by a bromine or chlorine atom. Thus, the bromination of calix[5]arene **8a** and calix[6]arene **8b** was carried out by the reflux them in CCl_4_ with a slight excess of N-bromosuccinimide and light irradiation (Scheme 3) [45,46].

The reflux of the pentabromocalix[5]arene derivative **9a** with HAA or dibenzoylmethane (HDBM) in the ionizing solvent 2,2,2-trifluoroethanol (TFE) under addition of several drops of HBr led to the formation of penta-1,3-diketone calix[5]arenes **10a** and **10b** (Scheme 3). Similarly, the interaction of **9b** with HAA gave the hexa-substituted calix[6]arene **10c**. The yield of the target product appeared to increase significantly with an increase of the size of the macrocycle (from 23% for calix[5]arene **10a** to 51% for calix[6]arene **10c**). The replacement of the TFE solvent with 1,1,1,3,3,3-hexafluoroisopropanol (HFIP) in the synthesis of compound **10c** also increased the yield of the reaction up to 62%. The authors supposed that the reaction proceeds in accordance with S*_N_*1 mechanism through the replacing of the halogen with a central carbon atom of the 1,3-diketone group. The structure of compound **10c** is characterized by the *cis*- (equatorial) arrangement of all 1,3-diketone fragments. The ^1^H NMR spectra of this compound in CDCl_3_ and DMSO-*d*_6_ solutions revealed no signals of the enol form.

Later, the calix[8]arene derivatives **12a**,**b** with one 1,3-diketone group attached to the methylene bridge of the macrocycle were obtained (Scheme 4) by the reflux of monochloro-substituted calix[8]arene **11** with HAA or HDBA in a mixture of chloroform and HFIP [47].

**Scheme 5:**
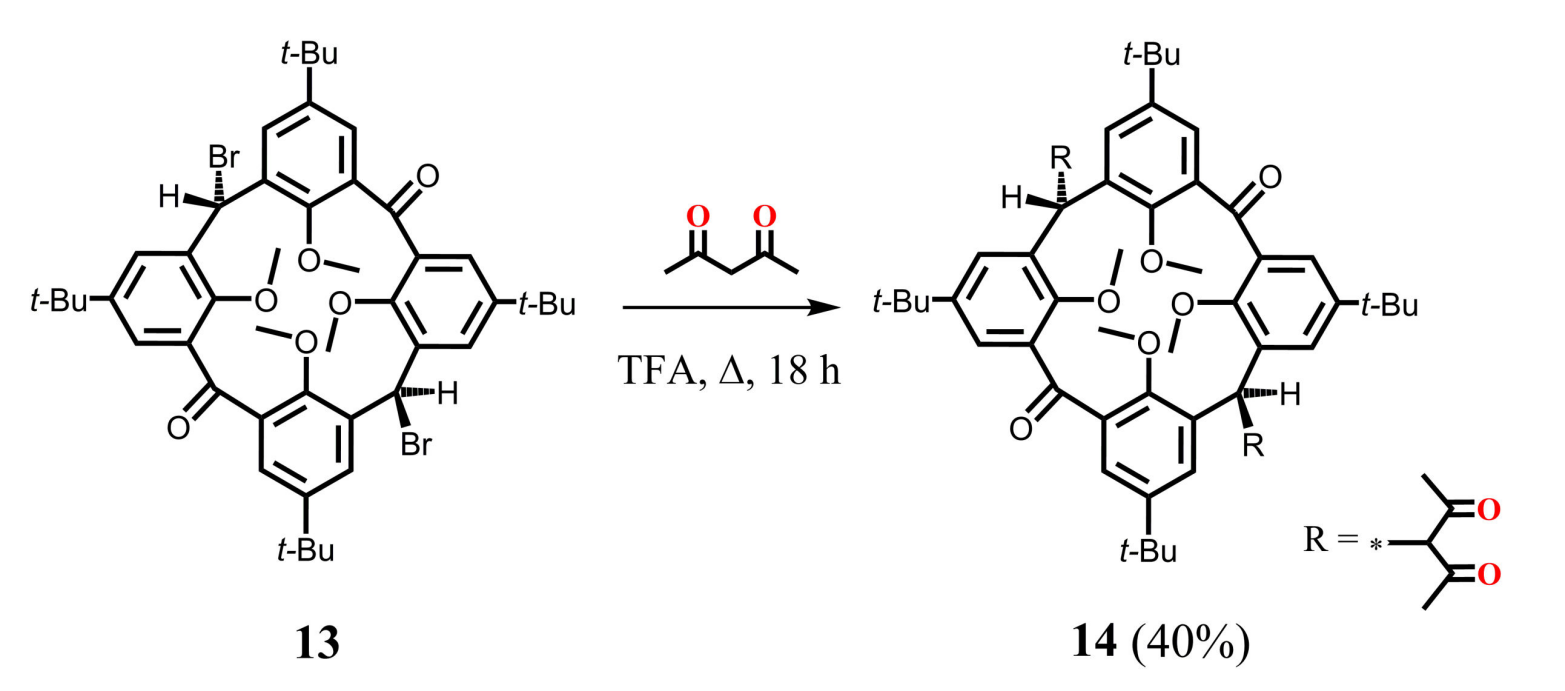
Preparation of bis-1,3-diketone derivative of calix[4]arene **14**.

The group of Biali S.E. succeeded in the synthesis of bis-1,3-diketone calix[4]arene derivative **14** (Scheme 5) [44] which was obtained by the reflux of dibromo-substituted calix[4]arene **13** bearing two carbonyl groups at the lower bridge with an excess of HAA in trifluoroacetic acid. A mutual *trans*-orientation of the chelate substituents at the macrocyclic platform appeared to be a peculiarity of the compound **14**. The absence of OH signals in ^1^H NMR spectrum of this compound indicates that only the keto form for 1,3-diketone fragments is realized in the CDCl_3_ solution.

**Scheme 6:**
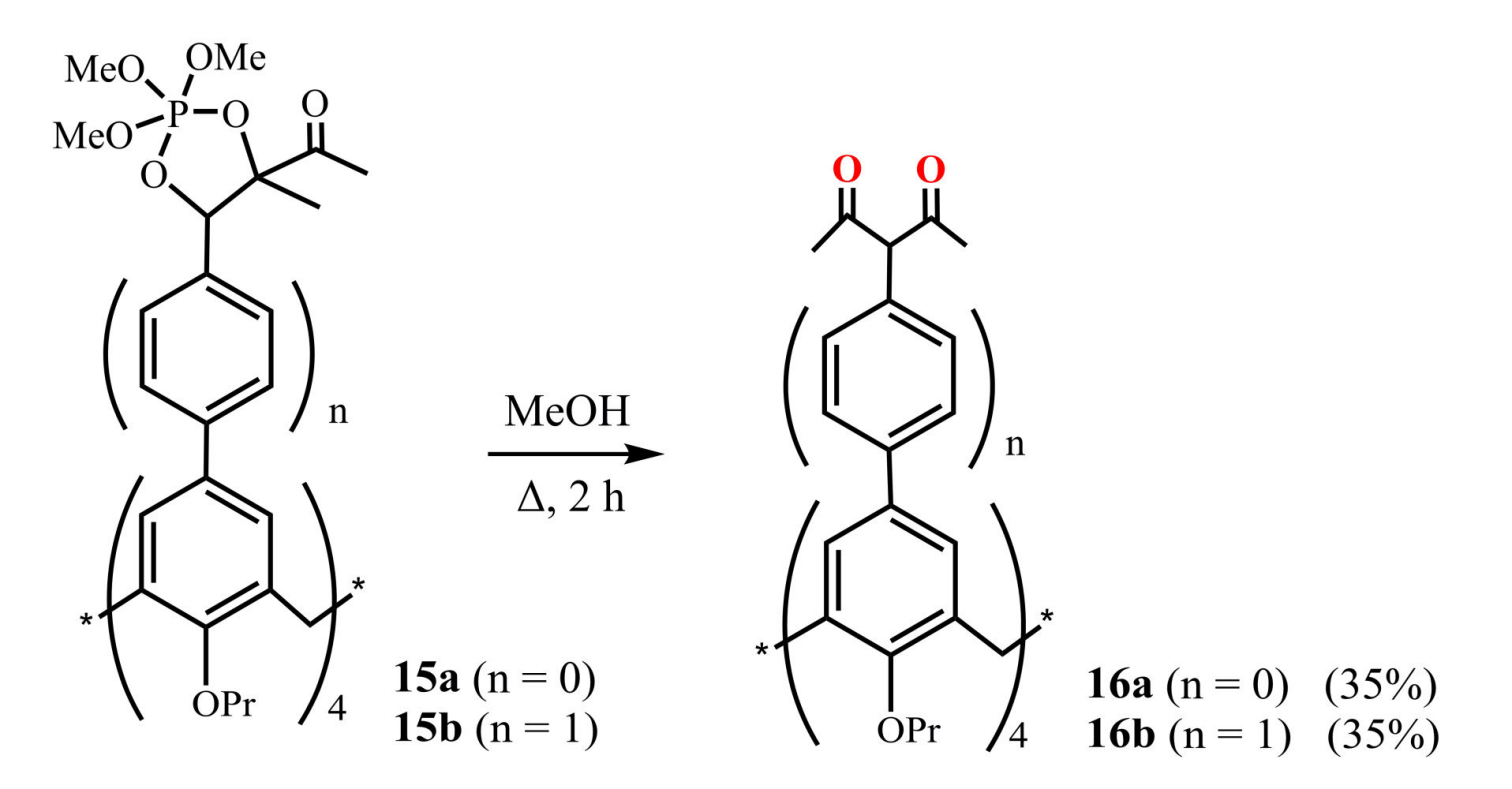
Preparation of tetra-1,3-diketone derivatives of calix[4]arene **16a**,**b**.

Jiang et al. (2014) [48] presented the synthesis of two types of calix[4]arenes **16a**,**b** bearing 1,3-diketone substituents at their upper rims and differentiating in the size of macrocyclic cavities (Scheme 6). For their preparation, 2,2,2-methoxy-4,5-dimethyl-1,3,2λ^5^-dioxaphosphole calix[4]arene derivatives **15a** and **15b** were refluxed in MeOH solution during 2 h giving the tetra-1,3-diketones **16a** and **16b** in 35% yield. The authors noted that the synthesis of **16b** was accompanied by a mixture of mono-, di-, tri-, and tetra-1,3-diketone calix[4]arene derivatives. Therefore, the target product was isolated using chromatography followed by recrystallization.

**Scheme 7:**
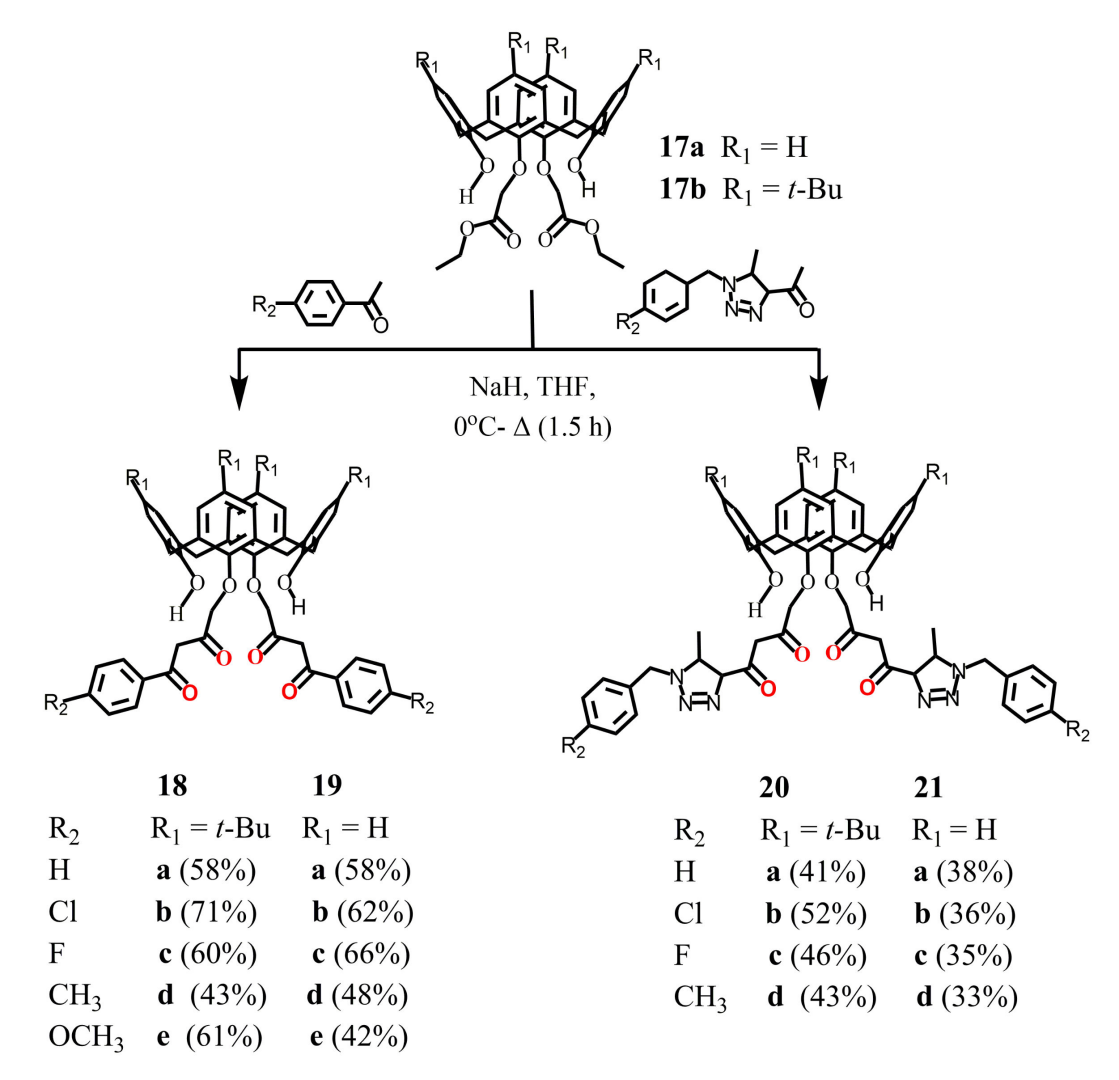
Synthesis of bis-1,3-diketone derivatives of calix[4]arene **18**–**21**.

Luo et al. (2014-2015) [49,50,51] reported the synthesis of bis-1,3-diketone calix[4]arene derivatives **18**–**21**, where the chelate groups were located at the lower rim of the macrocycle (Scheme 7). For this purpose, the Claisen condensation was carried out between diester calix[4]arene derivatives **17a**, **17b** and *para*-substituted acetophenones in the presence of 1.5 excess of NaH in THF. The isolated products were purified using chromatography column. The yield of bis-1,3-diketones **18a**–**e**, **19a**–**e** was 42–71%, and in the case of their analogues **20a**–**d** and **21a**–**d** containing triazole fragments, it turned out to be noticeably lower–33–52%. The resulting compounds have the *cone* isomeric form. The presence of OH-proton signals in the ^1^H NMR spectra of the synthesized bis-1,3-diketones **18**–**21** indicated a significant content of the enol form for these compounds. It should be noted that, unfortunately, our attempts to reproduce the synthesis of bis-1,3-diketone **18a** were unsuccessful, and no target product was found in the reaction mixture.

The side processes accompanying synthesis of poly-1,3-diketones can lead to a significant decrease in the yield of target products in comparison with the mono-derivatives. Sometimes it makes the synthesis of poly-1,3-diketones completely impossible. Moreover, several reaction centers and high molecular weights of calix[4]arenes complicates the synthesis of poly-1,3-diketones. Thus, an introduction of the previously prepared 1,3-diketone blocks into the macrocycle backbone may be considered as a more successful strategy for the obtaining of diketo-derivatives of calix[4]arenes. We have successfully used this approach earlier for the synthesis of tris-1,3-diketones based on the mesitylene platform [52], and the Shinkai group first applied it for the synthesis of bis-1,3-diketone calix[4]arene **2** [42]. In particular, the embedding of two or four 1,3-diketone groups onto upper rims of calix[4]resorcinarene, calix[4]arene and thiacalix[4]arene backbones was fulfilled by the substitution reactions of the previously prepared sodium salts of 1,3-diketones with the halogenmethylated macrocycles.

**Scheme 8:**
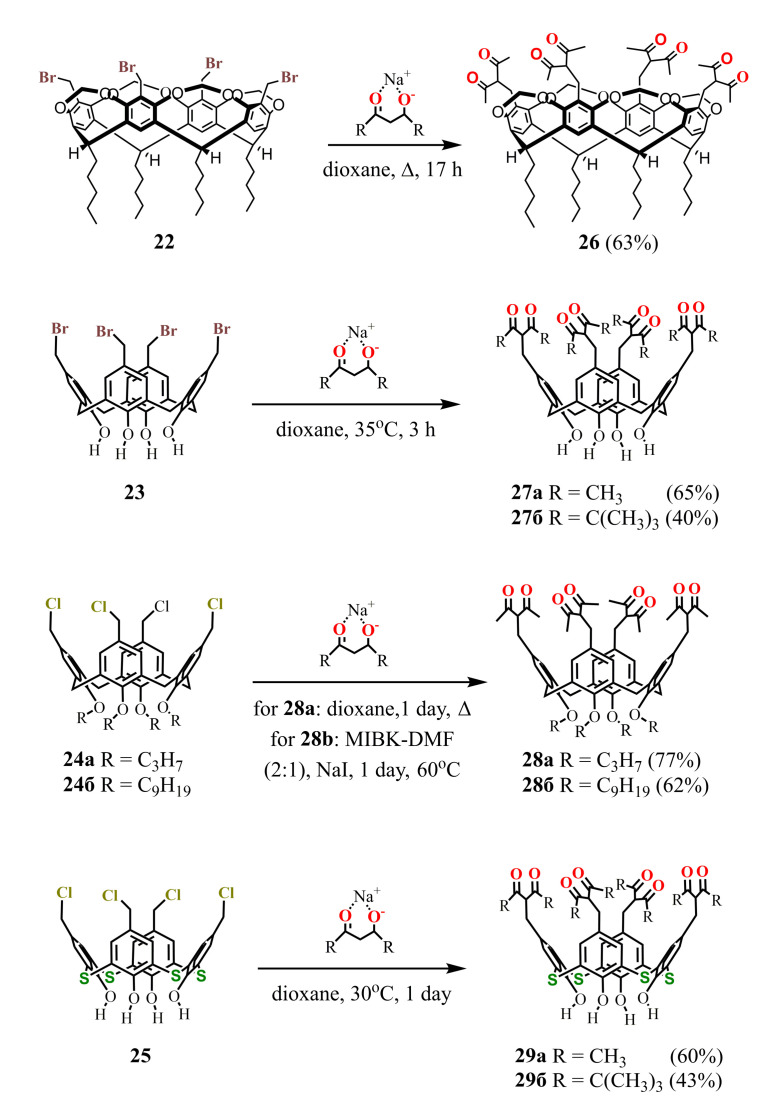
Synthesis of tetra-1,3-diketone derivatives of calix[4]resorcinarene **26**, calix[4]arene **27a**,**b**, **28a**,**b** and thiacalix[4]arene **29a**,**b**.

Thus, the reflux of tetra-bromomethylated calix[4]resorcinarene cavitand **22** or tetra-chloromethylated calix[4]arene **24a** with an excess of sodium acetylacetonate (NaAA) in dioxane resulted in the tetra-1,3-diketones **26** [53] and **28a** [54], respectively, in good yields (Scheme 8 ). However, in the case of calix[4]arene **24b** with long alkyl substituents, a generation of by-products due to the cleavage of diketone fragments was observed [55]. A lowering of the reaction temperature together with using of methyl isobutyl ketone (MIBK)–dimethylformamide (DMF) mixture instead of dioxane and addition of NaI enabled to avoid the by-products formation and to get the pure product **28b**.

Halogenomethylated derivatives of calix[4]arenes with alkyl substituents at the lower rim usually possess lower reactivity in comparison with their counterparts unsubstituted at the lower rim. Thus, the nucleophilic substitution of calix[4]arene **23** with NaAA or a sodium salt of dipivaloylmethane (NaDPM) proceeds even at room temperature and completes in a rather short time in contrast to the compound **24a** [54]. The chloromethylated analogue of **23** is known to be a highly reactive compound, especially under basic conditions [56,57]. It easily loses HCl to give the corresponding *p*-quinone methide which undergoes further transformations. The intensive reddening of the reaction mixture observed in 10-15 min after adding NaAA to the calix[4]arene **23**, is followed by the disappearance of color when the reaction is completed. These facts clearly indicate the formation of an intermediate quinone structure in the solution. The reaction with thiacalix[4]arene **25** proceeds in a similar way, giving the tetra-1,3-diketone derivatives **29a**,**b** [58] with the same yields as **27a**,**b**.

For the synthesis of bis-1,3-diketone calix[4]arene unsubstituted at the lower rim, the dibromomethylated derivative **31** was applied, being prepared at room temperature under interaction of calix[4]arene **30** with three equivalents of paraformaldehyde and HBr in glacial acetic acid solution in the presence of catalytic amount of Zn [55]. The addition of 1,3-diketone sodium salt to dibromomethylated calix[4]arene **31** was accompanied by the appearance of an intense pink-violet color caused apparently by the formation of *p*-quinone methide intermediates in the solution. At the end of reaction, the target bis-1,3-diketones **32a**–**c** were isolated with 52–60% yields [55].

**Scheme 9:**
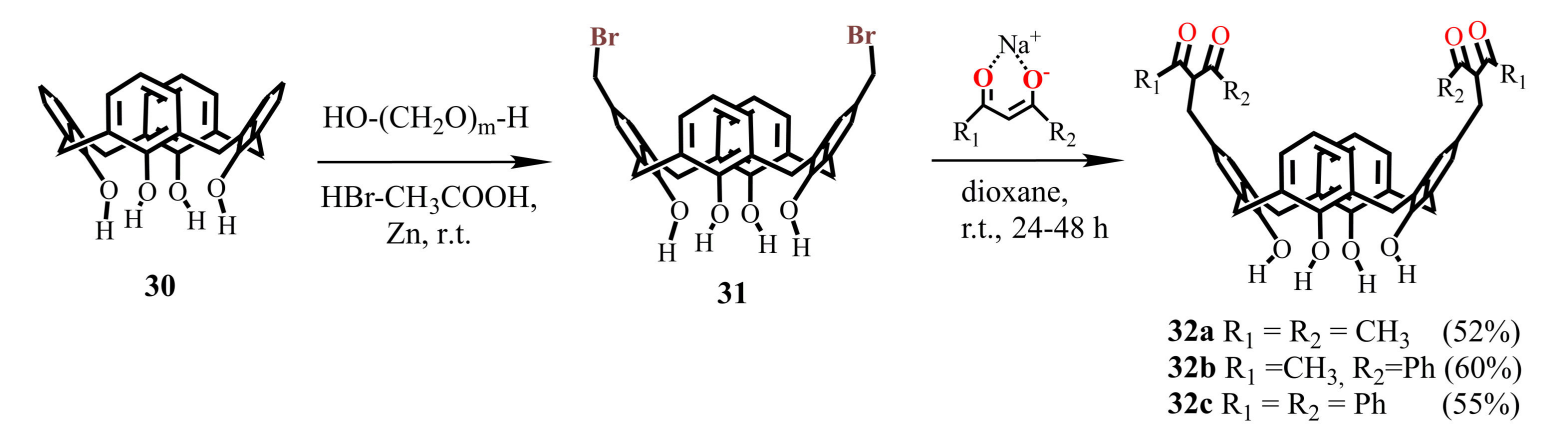
Synthesis of bis-1,3-diketone derivatives of calix[4]arene **32a**–**c**.

**Scheme 10:**
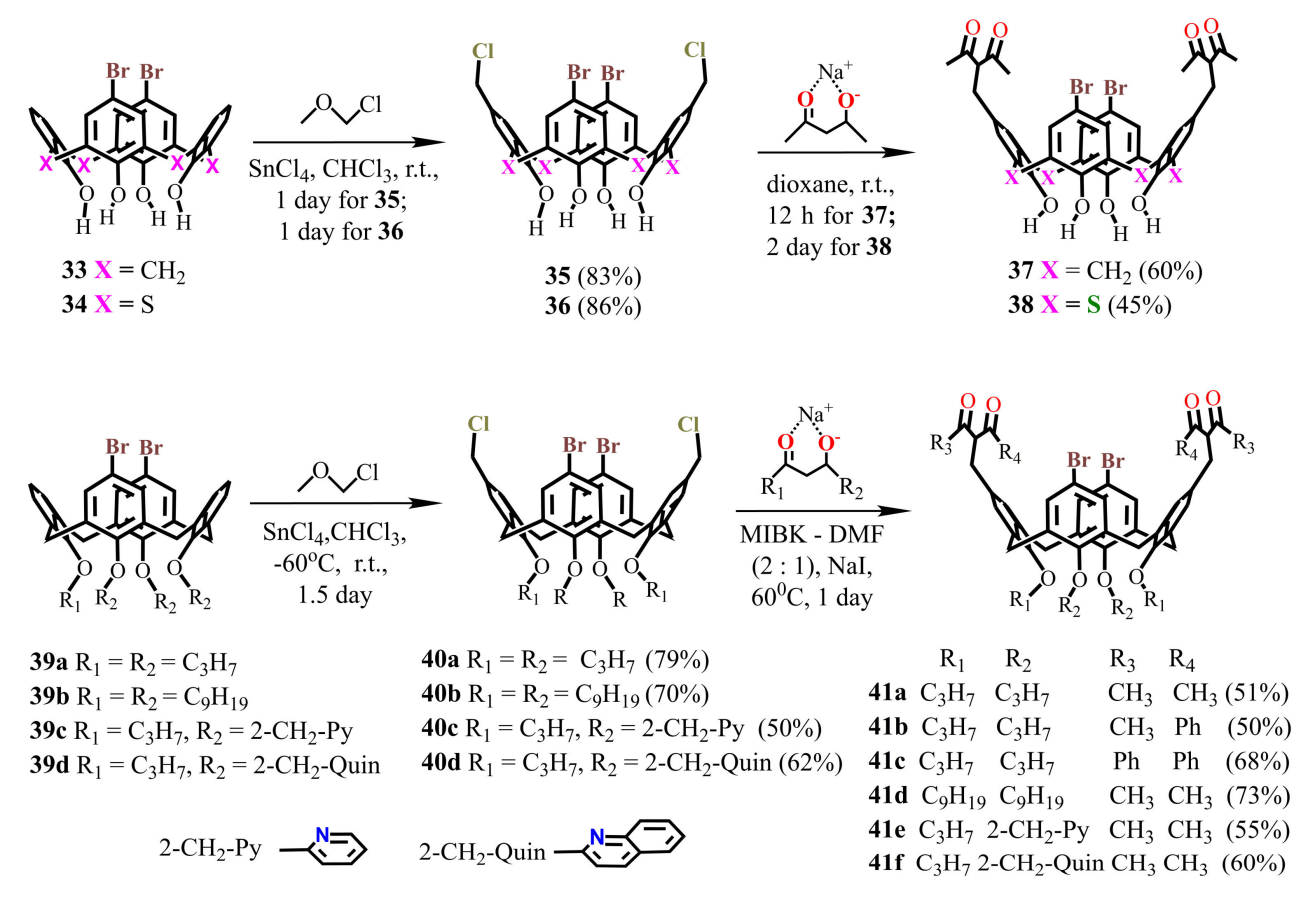
Synthesis of dibromo-bis-1,3-diketone derivatives of calix[4]arene **37**, **41** and thiacalix[4]arene **38**.

The approach represented by Scheme 9 cannot be applied for the synthesis of bis-1,3-diketone calix[4]arene derivatives having substituents at the lower macrocyclic rim due to low reactivity of the substituted calix[4]arenes. However, the halogenmethylation of such calix[4]arenes can be performed by the addition of an excess of chloromethyl methyl ether and SnCl_4_. In the case of thiacalix[4]arene, this reaction proceeds only for the macrocycle unsubstituted at the lower rim. On the whole, the possible formation of both structural isomers and differently substituted by-products under the excess of the reagents complicates the controlled preparation of the distally substituted dichloromethylated derivatives. In this connection, a more general approach has been developed, based on the usage of dibromo-calix[4]arenes **33**, **34** and **39a**–**d** (Scheme 10).

The synthesis of compounds **33** [59] and **34** [60] was carried out by means of protection and deprotection procedures using a benzoyl group, as well as by introduction of bromine atoms at the upper rim of calix[4]arene applying Br_2_. Dibromo-calix[4]arenes **39a,b** with alkyl substituents were obtained by the procedures of dialkylation, bromination, and alkylation of the remaining hydroxyl groups. Calix[4]arenes **39c,d** with pyridine or quinoline fragments at the lower rim of the macrocycle [61] were synthesized in a similar way, where the pyridine or quinoline groups were introduced at the first stages of the synthesis. The reaction of these compounds with an excess of chloromethyl methyl ether and SnCl_4_ yielded dichloromethylated derivatives **35**, **36** and **40a**–**d** which, being reacted with sodium salts of 1,3-diketones, gave the target bis-1,3-diketones **37**, **38** and **41a–f** with good yields (Scheme 10).

Thus, all 1,3-diketone derivatives presented in the literature were obtained on the basis of “classical” calix[4]arenes functionalized at both upper and lower rims. There are also several examples of calix[4,5,6 and 8]arenes with chelating groups attached to the methylene bridges of these macrocycles. To obtain all these compounds, various synthetic methods were studied. Many of these methods, however, have a rather limited application and often are accompanied by a low yield. We have developed a more general and efficient approach to the preparation of poly-1,3-diketones, based on the reaction of nucleophilic addition of 1,3-diketones to the halogenmethyl derivatives of calix[4]arene. Applying this approach, we succeeded in obtaining of a wide range of bis- and tetra-1,3-diketone derivatives of calix[4]arene, thiacalix[4]arene, and calix[4]resorcinarene, including bifunctional bis-1,3-diketone calix[4]arene derivatives.

## 3. Keto-Enol Tautomeric Transformation of 1,3-Diketone calix[4]arene Derivative

**Scheme 11:**
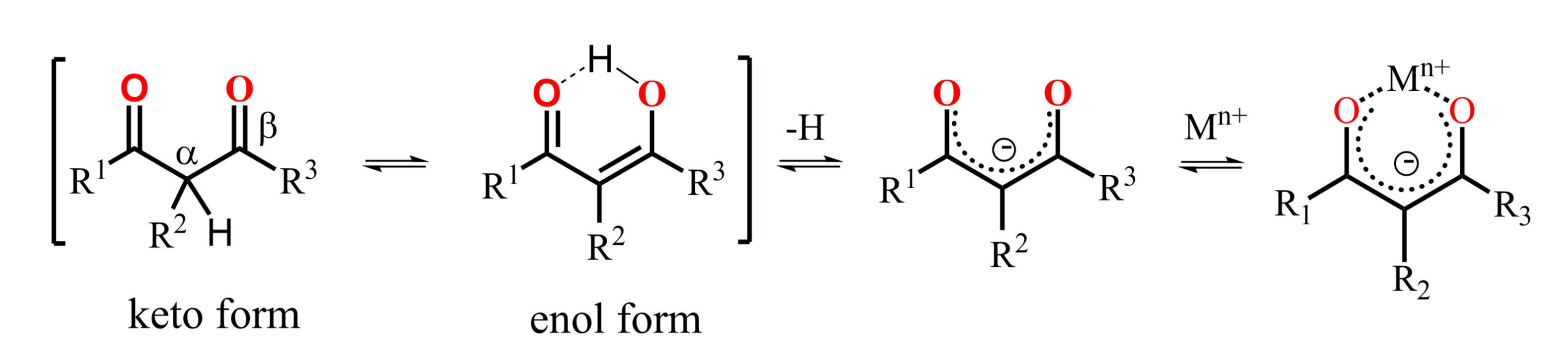
Keto–enol transformation of 1,3-diketones, 1,3-diketonates and their complexes with metal ions.

A keto-enol tautomeric transformation of 1,3-diketones represented in Scheme 11 is the most widely documented property of the 1,3-diketone derivatives [62,63,64,65,66]. The extent of enol form of 1,3-diketones in solutions tends to increase under dilution of the latter or under decreasing a solvent polarity [23,62,67]. Literature data reveal great impact of both α- and β-carbon substitution on the keto-enol transformation, because thermodynamic stabilization of the enolate form requires the charge delocalization, which is facilitated by the planar localization of both β-carbon substituents and keto-groups in the *cis*-localization (Scheme 11). Indeed, the bulky α-carbon substituents destabilize the *cis*-enol form [68], while the greater bulkiness of β-carbon substituents exemplified by methyl, *tert*-butyl and phenyl moieties enhance the extent of the enolic form. The pronounced enhancement of this form for phenyl-substituted 1,3-diketone derives from facilitating the π-delocalization around the enol ring [62].

**Figure 1:**
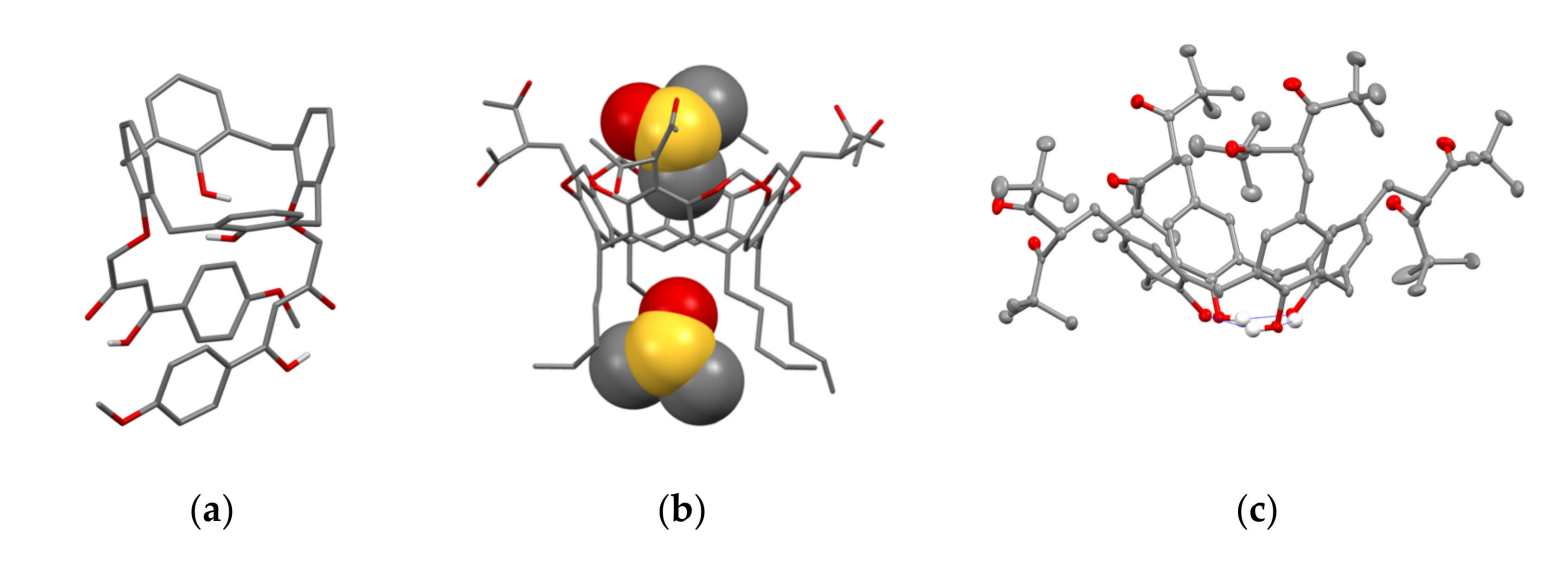
The molecules **19e** (**a**), **26** (**b**)and **27b** (**c**) in the crystal. Molecule of **26** (**b**) with two DMSO molecules is represented in space-filled model. Hydrogen atoms are omitted for clarity. Redrawn from reference [49] (**a**). Reproduced from Ref. [53] (**b**) and [58] (**c**) with permission from the Centre National de la Recherche Scientifique (CNRS), the Royal Society of Chemistry and the Elsevier, correspondingly.

Literature data analysis reveals an impact of the embedding mode of 1,3-diketo-groups onto cyclophanic backbones on the keto-enol transformations. In particular, the lack of enolic form is observed when 1,3-diketo-groups are embedded via bridging methylene-group (**14** in Scheme 5), while the enolic form is predominant when 1,3-diketone moieties are embedded through the lower rim of calix[4]arene (**18**–**21** in Scheme 7). In particular, 1,3-diketone **19e** in crystalline state was revealed in enol form (Figure 1a) [49]. The contribution of enolic form is also high (30–50%) under the upper-rim substitution of acetylacetone groups (**27**–**29** in Table 1). The embedding of acetylacetone moieties onto calix[4]resorcinarene cavitand **26** (Figure 1b) via α-carbon substituted methylene groups significantly restricts the keto-enol transformation which results in the predominance of the keto-form in crystalline state of **26** and low extent of the enolic form (~5–10%) in CDCl_3_-based solution [53].

The embedding of two or four 1,3-diketo-groups onto the upper rim of calix[4]arene is followed by the interference between the different tautomeric forms. For example, keto-keto (50%), keto-enol (30%) and enol-enol (20%) are revealed in solutions of bis-1,3-diketone calix[4]arene **37** [55]. It is also worth noting the specificity of keto-enol tautomerism for 1,3-diketo-groups embedded onto the calix[4]arene platform. This is well exemplified by the decreased extent of enol form (<0.2%) under the substitution of methyl groups on phenyl or *t*-Bu ones in the β-position [55] (Table 1), since the keto-enol transformation is restricted by the steric hindrance illustrated by the structure of **27b** in Figure 1c.

## 4. Complex Formation of 1,3-Diketone Calix[4]arene Derivatives with Metal Ions

The enolic form derived from the keto-enol transformation in alkaline media can form carbanions or enolates, while the latter can be stabilized by the complex formation with metal ions (Scheme 11). The presence of the pre-organized donor oxygen atoms of enolate form provides the efficient chelating of both *d*- and *f*-transition metal ions [23]. The complex formation of the commonly applied 1,3-diketones results in the formation of tris-chelates, which saturate the coordination sphere of *d*-transition metal ions, while the high coordination numbers of lanthanide ions provide the reason for remaining one or two coordination sites unoccupied under their tris-chelation with 1,3-diketonates [23]. The combination of two or greater number of 1,3-diketone moieties within one molecule restricts their abilities to tris-chelation, thus, raising the necessity to ternary complex formation [25], which makes such complexes promising basis for a substrate-induced luminescent answer.

**Figure 2:**
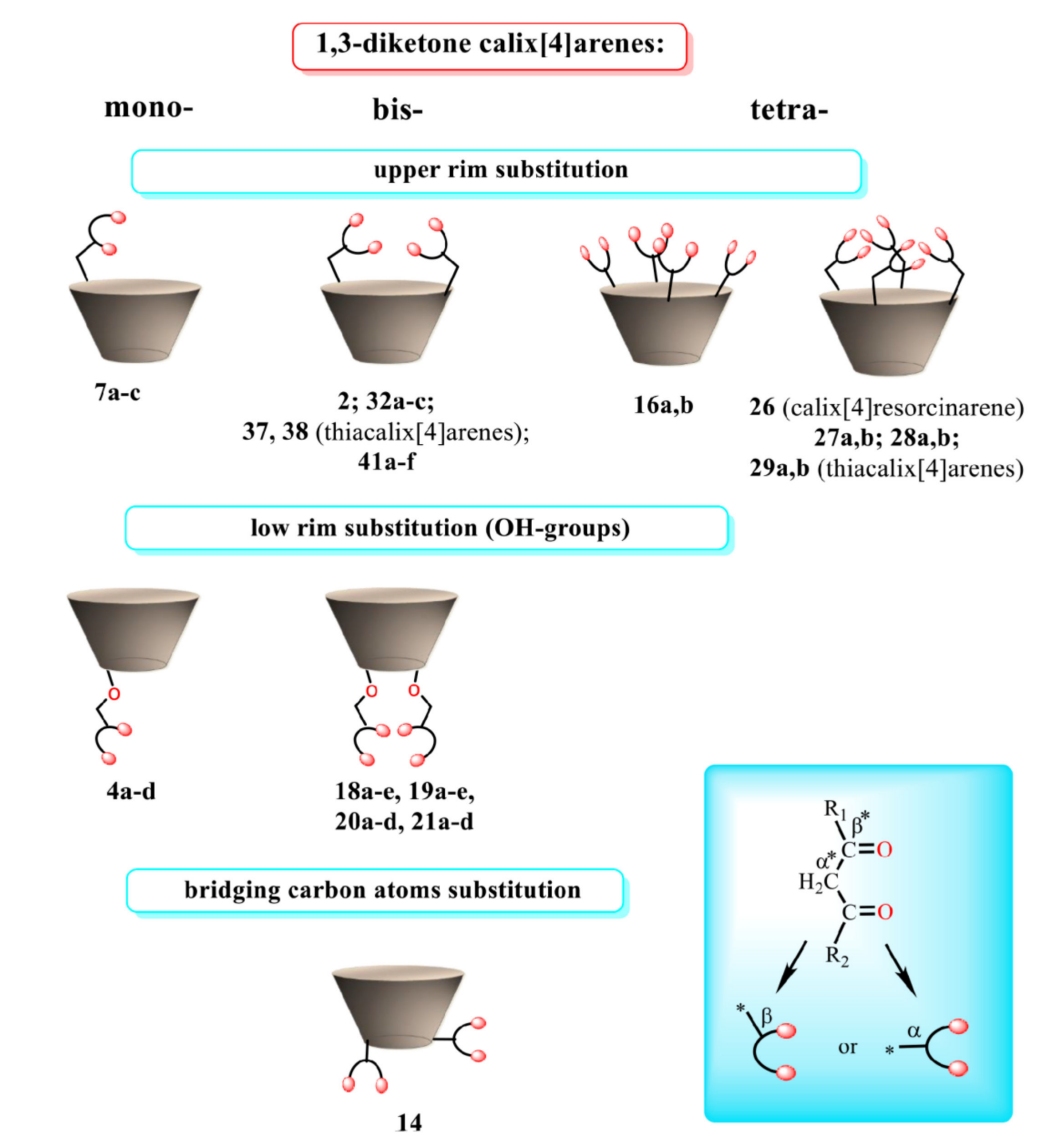
Schematic presentation of 1,3-diketone calix[4]arenes with different embedding modes of 1,3-diketone groups onto cyclophanic backbone.

The various ways of embedding of 1,3-diketone moieties onto calix[4]arene backbone are schematically illustrated in Figure 2. It is obvious that the embedding mode of 1,3-diketo-groups is of great impact on their ability to coordinate metal ions through the bis-chelation, since the latter results in more tight metal ion coordination than a mono-chelation by single 1,3-diketonate group. It is worth anticipating that a bis-chelation of metal ions by rigidly oriented 1,3-diketone groups in structures **16a**,**b** can be realized only in sandwich-like dimeric metal complexes, while the embedding of the 1,3-diketone-groups via methylene bridge onto calix[4]arene (structure **2** in Figure 2) facilitates their ability to both intramolecular and intermolecular chelating of metal ions. However, literature data on the complex formation of calix[4]arene derivative **2** with metal ions is exemplified by copper ions only, where the sandwich-like 2:2 complexes based on the bis-chelation of Cu^2+^ ions with 1,3-diketonate groups of two calix[4]arene ligands has been revealed [42].

**Figure 3:**
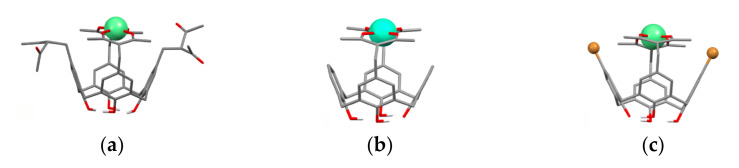
Bis-chelated coordination of Tb^3+^ ion by tetra-1,3-diketone **27a** (**a**) and bis-1,3-diketone **32a** (**b**) and **37** (**c**) calix[4]arenes. The structures are obtained after SPARKLE/PM7 optimization with the use of MOPAC 2012 software. Reproduced from Ref. [54] (**a**), [59] (**c**) and redrawn from reference [55] (**b**) with permission from the Elsevier, the Centre National de la Recherche Scientifique (CNRS) and the Royal Society of Chemistry.

It is worth assuming that 1,3-diketonate groups of the calix[4]arenes upper-rim substituted by both two and four 1,3-diketone groups **27a**,**b**, **28a**,**b, 32a**–**c** and **41a**–**f** can provide only 1:1 bis-chelation of metal ions. The assumption has been proved by both experimental results and quantum-chemical calculations [54,55,69]. The bis-chelation of terbium ions by 1,3-diketonates is more thermodynamically favorable than their coordination with the lower phenolic/phenolate rim of both tetra- (**27a**) [54] and bis-acetylacetone-substituted calix[4]arenes **32a**, **37** [55,59] as it is illustrated by Figure 3. Thus, the presence of the lower phenolic rim doesn’t affect the coordination of Tb^3+^ ion with 1,3-diketonates embedded at the upper rim of calix[4]arene backbone [54,55].

**Figure 4:**
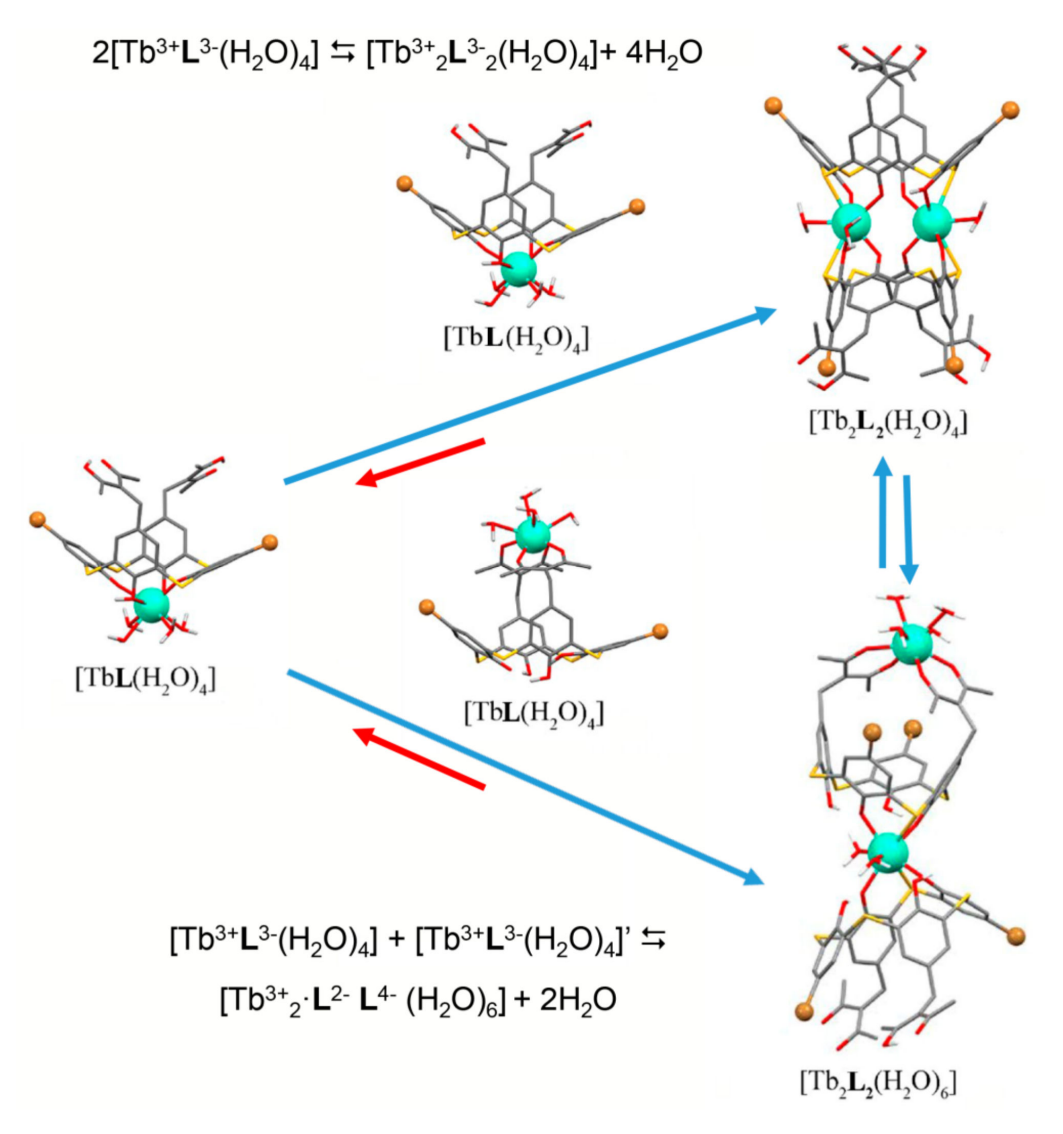
Schematic presentation of the equilibration between monomeric complexes [Tb**L**(H_2_O)_4_] and dimeric complexes [Tb_2_**L**_2_(H_2_O)_4_] and [Tb_2_**L**_2_(H_2_O)_6_] (**L** = **38**) in the DMF solutions.

The opposite tendency is observed for its thia-analogue [58,60,69]. The complexing capacity of the lower thiacalix[4]arene’s phenolic rim is much higher than that of its classical counterpart [71,72,73]. This makes the coordination of Tb^3+^ ion via lower thiacalix[4]arene’s phenolic rim more thermodynamically favored than its bis-chelation with two diketonate groups [69]. The lower rim coordination is enforced by the dimerization of the 1:1 complexes in accordance with Figure 4 which illustrates the coordination modes of Tb^3+^ ions with dibromo-bis-1,3-diketone thiacalix[4]arene **38**. The similar coordination is observed for the tetra-1,3-diketone thiacalix[4]arenes **29a**. However, the comparative analysis of both steady state and time resolved luminescence of terbium complexes with thiacalix[4]arene and its 1,3-diketone derivative in DMF indicates that the bis-chelation via the upper rim contributes to the complex formation [60,69].

**Figure 5:**
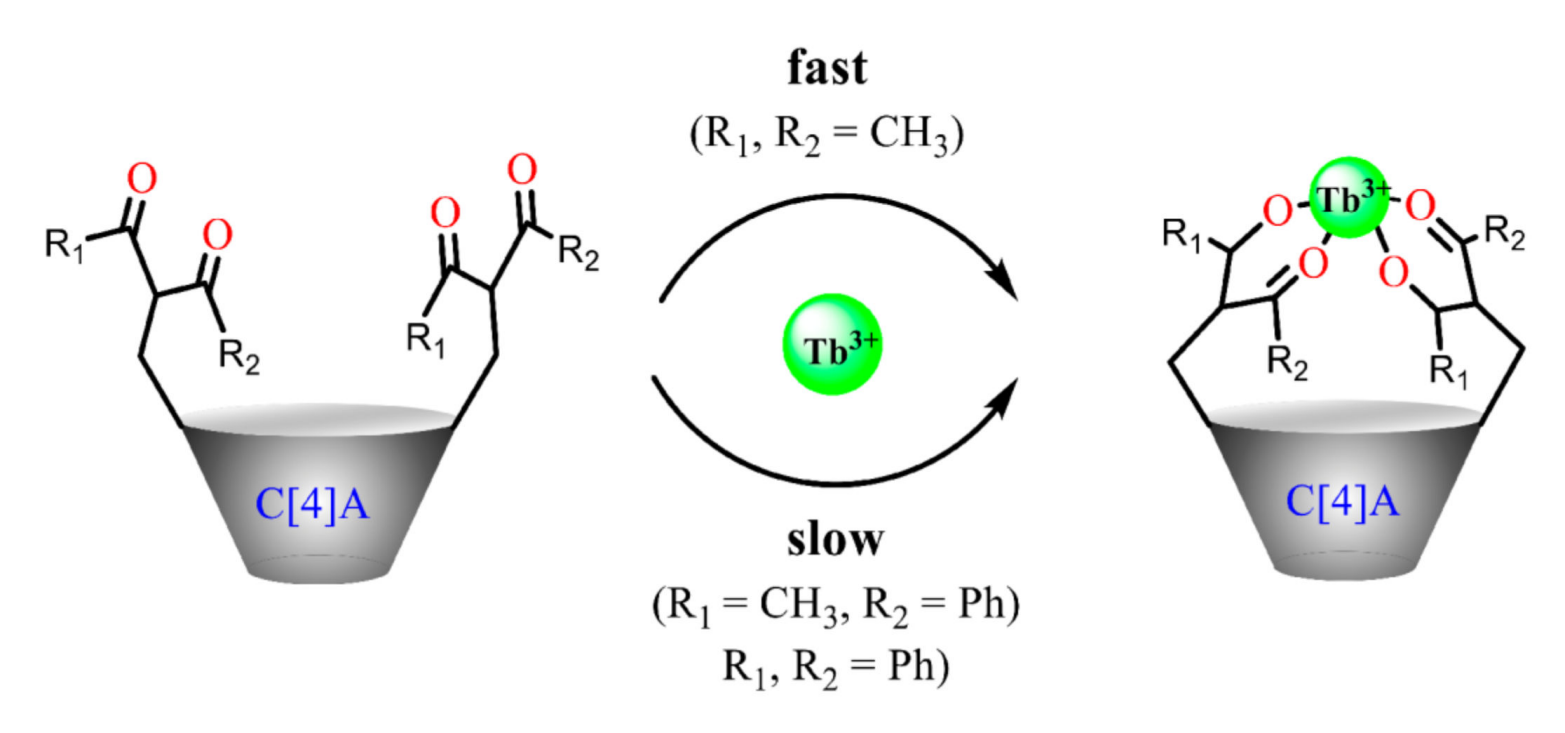
Cartoon illustration of the substituents effect on the complex formation of 1,3-diketone calix[4]arenes with Tb^3+^ Reproduced from Ref. [55] with permission from the Elsevier, the Centre National de la Recherche Scientifique (CNRS) and the Royal Society of Chemistry.

As it has been already noted the benzoylacetone moieties embedded onto calix[4]arene platform exhibit the restricted keto-enol transformation [59]. Thus, the complex formation of the calix[4]arenes bearing benzoylacetone moieties (**32a**–**c** in Scheme 9) with terbium ions is slowed down and comes to the equilibrium conditions within one day, while the complex formation extent is still insignificant within one day for the diphenyl substituted 1,3-diketone derivatives Figure 5 [55]. This tendency differs from the common 1,3-diketones, where β-carbon phenyl-substitution results in the additional stabilization of the enol form through the coupling of diketonate ring with the aromatic substituents.

**Figure 6:**
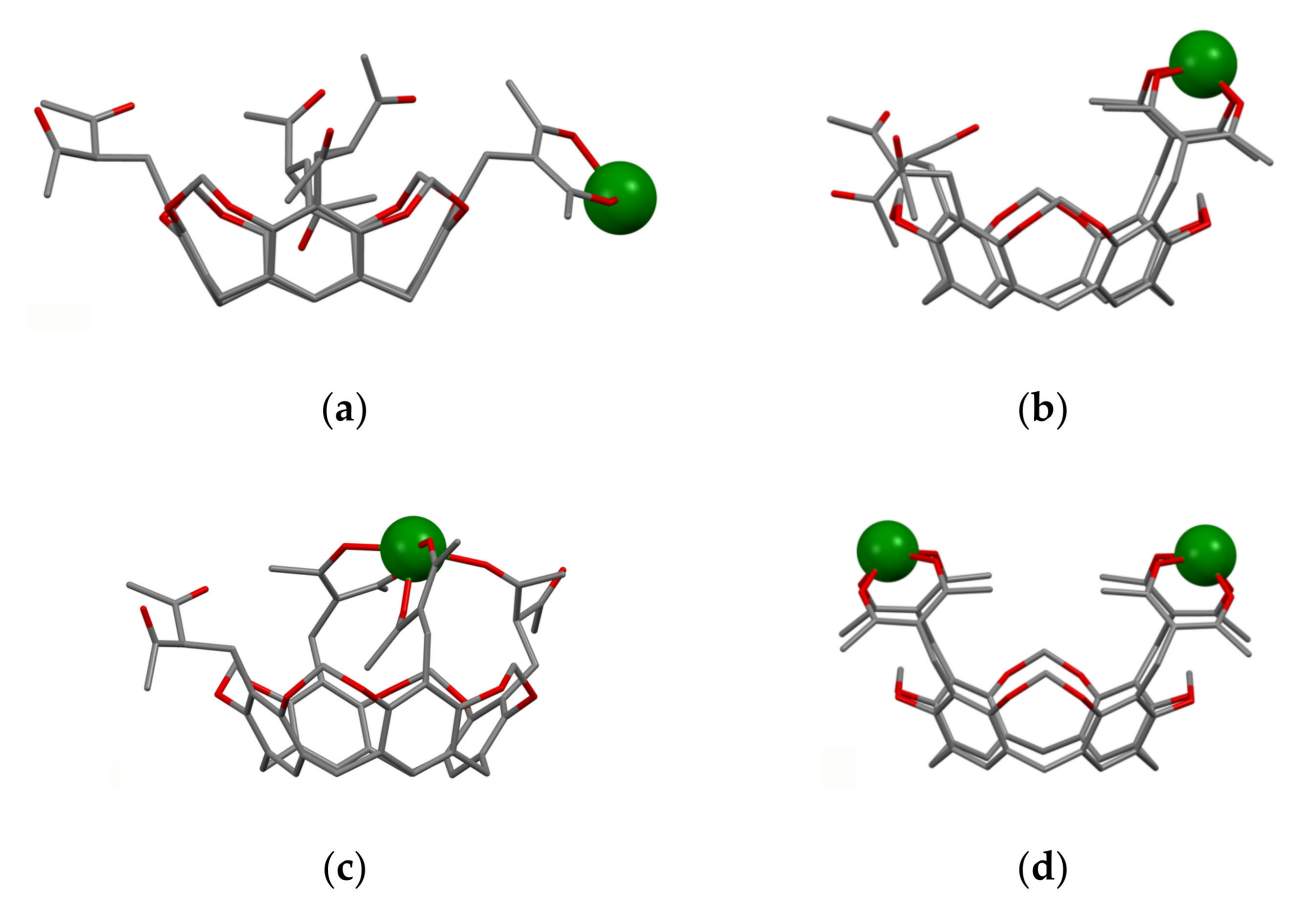
Possible modes of binding of Tb^3+^ cations to molecule **26** with participation of one (**a**), two (**b**), three (**c**) and four (**d**) 1,3-diketone groups (lower rim alkyl substituents are omitted)The structures are obtained after PM6 optimization of starting structures built on the basis of single-crystal X-ray data for molecule **26**. Reproduced from Ref. [53] with permission from the Centre National de la Recherche Scientifique (CNRS) and the Royal Society of Chemistry.

The embedding of 1,3-diketone moieties onto rigid structure of the calix[4]resorcinarene cavitand through the α-carbon substitution results in greater effect on keto-enol transformation than the embedding onto calix[4]arene or thiacalix[4]arene backbones. The upper rim of the cavitand is wider than that of calix[4]arene, which provides the opportunity of the binding of two lanthanide ions through the bis-chelation with 1,3-diketonate groups of calix[4]resorcinarene cavitand. However, the formation of the thermodynamically more stable 2:1 complex (Figure 6d) is kinetically retarded and undergoes through the 1:1 complex formation (Figure 6a–c) [53].

## 5. Lanthanide-Centered Luminescence Sensitized by the Cyclophanic 1,3-Diketones

The peculiarity of a lanthanide-centered luminescence derives from the fact that the radiation decay occurs from the singlet excited state, which is mainly fed by ligand-to-metal energy transfer, although the energy of the excited state is independent on the ligand environment [74]. Thus, design of optimal ligands providing efficient antenna-effect on lanthanide-centered luminescence should be based on tuning of ligand-centered triplet level for the optimal ligand-to-metal energy transfer.

The structure variation of the substituents neighboring to the diketo-group is the well-known tool for the triplet level energy optimization. This is well exemplified [23] by the series of 1,3-diketones, where substitution of methyl substituents on benzyl or thenoyl moieties modifies the triplet level, which, in turn, enables to sensitize the green or red luminescence of Tb^3+^ or Eu^3+^. The similar effect of substituents has been revealed for the differently substituted 1,3-diketone derivatives of calix[4]arenes (**32a**–**c [55]**, **41a**–**c [59]** on Table 1). In particular, the lower energy of the triplet level of the benzoylacetone-substituted calix[4]arenes (**32b, 41b**) versus the acetylacetone-substituted ones (**32a, 37, 41a,d**) is the reason for the different antenna effects on the Tb^3+^-and Yb^3+^-centered luminescence. It is worth noting that the benzoylacetone-substituted ligand in greater extent than its acetylacetone-substituted counterpart sensitizes the NIR luminescence of Yb^3+^, while the acetylacetone-substituted ligand is better antenna for Tb^3+^-luminescence than its benzoylacetone-substituted counterpart [55]. However, the complex formation of benzoylacetone-substituted calix[4]arene with Ln^3+^ ions is greatly retarded due to the above mentioned effect of the α-carbon substituents on the keto-enol tautomeric equilibrium of the 1,3-diketone derivatives of calix[4]arenes. This, in turn, restricts the antenna-effect of benzoylacetone- or dibenzoylmethane-substituted calix[4]arenes on the Ln^3+^-centered luminescence.

**Figure 7:**
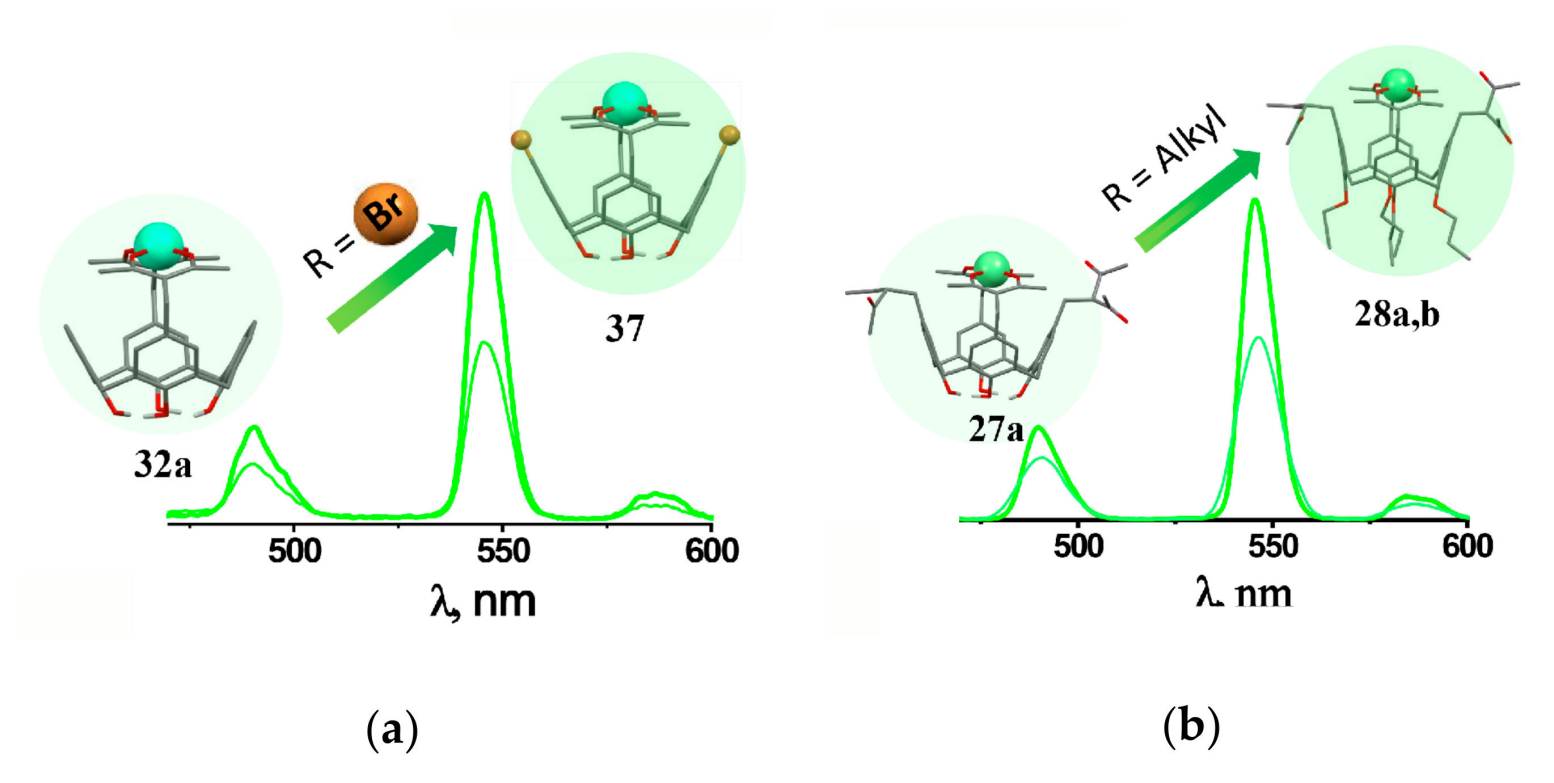
Cartoon illustration of the effect of lower rim (**a**)and upper rim (**b**) substituents on the Tb^3+^-centered luminescence of [Tb^3+^L] complexes with differently substituted 1,3-diketone calix[4]arenes. Reproduced from Ref. [59] (**a**) with permission from the Elsevier.

The energy stock from the excited state through the radiationless decay is the well-known reason for quenching of lanthanide-centered luminescence. Thus, the restricted vibrational movement of the inner-sphere ligands lowers the radiationless decay contribution. The fluorination or bromination of methyl or phenyl moieties is the well-known prerequisite for rigidifying of the inner-sphere environment of lanthanide ions [23,35,75]. The upper-rim bromination of 1,3-diketone derivatives of calix[4]arene (**37**, **41a**–**f**) decreases the radiationless decay, which exemplifies the so-called heavy atom effect [59] (Figure 7a).

It is worth assuming that the pre-organization of the chelating groups at the cyclophanic backbone is one more prerequisite for rigidifying of the inner-sphere environment of lanthanide ions. This is manifested by the great values of the excited state lifetime values measured for the 1:1 terbium complexes, although the hydration number (q) is above 4, which is higher than for terbium tris-acetylacetonate (q = 2) [35,36]. The aforesaid is worth correlating with the rigid inner-sphere environment resulted from the chelation of Tb^3+^ ion via two diketonate groups embedded onto the calix[4]arene platform. Moreover, the lower rim functionalization exemplified by the alkoxy-substitution of the lower rim hydroxyls (**27a**, **28a**,**b**) enhances both Tb^3+^- and Yb^3+^-centered luminescence (Figure 7b) [54]. The tendency derives from the conformation flexibility of the non-substituted calix[4]arene due to disturbance of the intramolecular hydrogen bonding at the phenolic/phenolate rim, in turn, resulted from its deprotonation in the basified conditions required for the diketone-to-diketonate transformation, while the alkoxy-substituents restrict the conformation flexibility.

It is worth noting the different effects of the bis-bromination of 1,3-diketone cyclophanic derivatives based on calix[4]arene and thiacalix[4]arene platforms on their ability to sensitize the Tb^3+^-centered luminescence [59,60]. As it has been mentioned above, the dibromination of calix[4]arene scaffold (**37**) enables to sensitize the Tb^3+^-centered luminescence due to the so-called “heavy atom effect” decreasing the radiationless decay through rigidifying of intramolecular vibration and stretching movements. The bis-bromination of the 1,3-diketone thiacalix[4]arene (**38**) results in the quenching of the steady-state luminescence of its Tb^3+^ complexes, while it leads to the insignificant effect on the excited state lifetime of the luminescence (Table 1).

The difference in the coordination modes of Tb^3+^ in the complexes with 1,3-diketone calix[4]arene and its thia-analogue explains the aforesaid difference (Figure 3 and Figure 4). Indeed, in the case of thiacalix[4]arene ligands the Br-substituents affect the triplet level energy (Table 1) [60], since the inductive and resonance effect of the substituents influence the ability of the ligand to feed the *f*-centered excited state through the Dexter mechanism. The decreased gap between the feeding and excited energy levels facilitates the back energy transfer, which, in turn, restricts the antenna effect of the thialix[4]arene ligand bearing the Br-substituents in comparison with the luminescence of the non-substituted counterpart.

**Figure 8:**
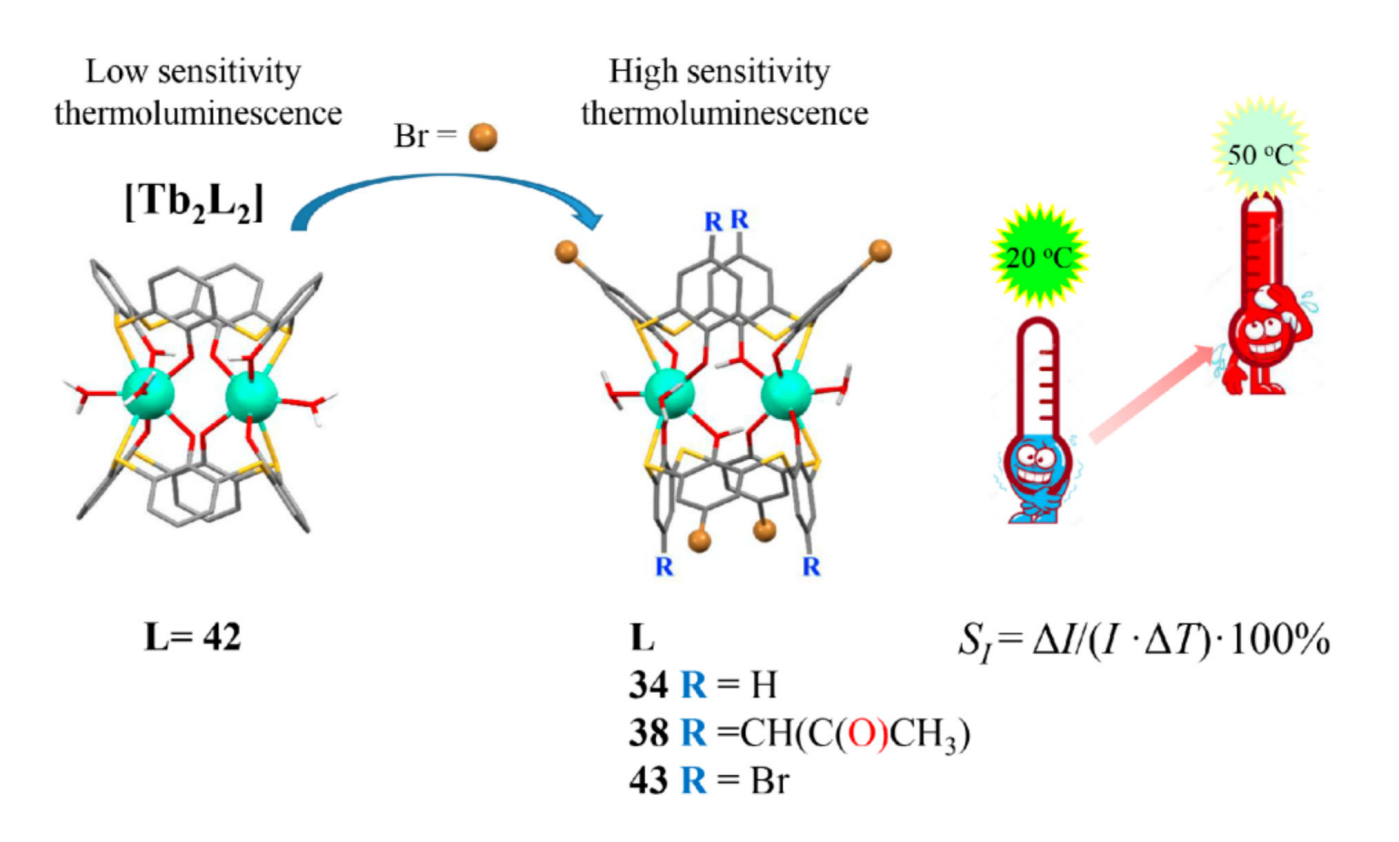
Cartoon illustration of the impact of the bromo-substitution on the thermal sensitivity of Tb^3+^-centered luminescence for dimeric complexes with the thiacalix[4]arene derivatives Redrawn from reference [60] with permission from the Royal Society of Chemistry.

The steady state luminescence intensity tends to linear decrease under the heating within the physiological temperature range (20–50 °C) for the dimeric Tb^3+^ complexes with dibromo- (**34** and **38**) and tetra-bromo-substituted (**43**) thiacalix[4]arenes, while the insignificant temperature-induced quenching is revealed for the isostructural complexes with the non-substituted thiacalix[4]arene **42** (Figure 8). The quenching extent is up to 50 % for the complexes with dibromo- and tetrabromo-substituted counterparts, and the relative thermal sensitivity *S_I_* (Table 1) values are greater than similar values documented in literature [32,76,77,78,79] for the terbium complexes with another ligands. Thus, the terbium complexes with the bromo-substituted thiacalix[4]arenes provide a good basis for developing of molecular thermometers.

It is also worth noting that high coordination number of the terbium complexes is the prerequisite for further ternary complex formation with chelating substrates resulting in the luminescence answer [37], although the poor water solubility of the complexes restricts their applicability in sensing. Moreover, the embedding of pyridyl or quinolyl groups at the lower rim of calix[4]arene bearing 1,3-diketone groups at the upper one provides the basis for heterometallic complex formation with Tb^3+^ ions bis-chelated by β-diketonates at the upper and copper ions coordinated with N-donor atoms at the lower rims of calix[4]arene (**41e**,**f**) [61]. The lower rim coordination of copper ions is followed by the quenching of Ln^3+-^centered luminescence through the donor-acceptor energy transfer mechanism [80,81]. However, the potential of the developed terbium complexes in sensing can be highlighted only after their transformation into water dispersible nanoparticulate form.

## 6. Nanoparticulate Forms of Cyclophanic 1,3-Diketonate Complexes for Sensing and Imaging

The transformation of 1,3-diketonate lanthanide complexes into water dispersible forms is the well-known prerequisite for their applicability in sensing and imaging. The transformation can be achieved through different synthetic modes, which are discussed herein from a viewpoint of their usability for conversion of the lanthanide complexes with cyclophanic 1,3-diketonates into water dispersible nanoparticulate forms. The doping of the complexes into polymeric water dispersible nanoparticles commonly undergoes through the blending of the complexes with monomers followed by the stimuli-triggered polymerization. This route is convenient for embedding of 1,3-diketonate lanthanide complexes into polymeric films and nanoparticles [28,82,83,84].

**Figure 9:**
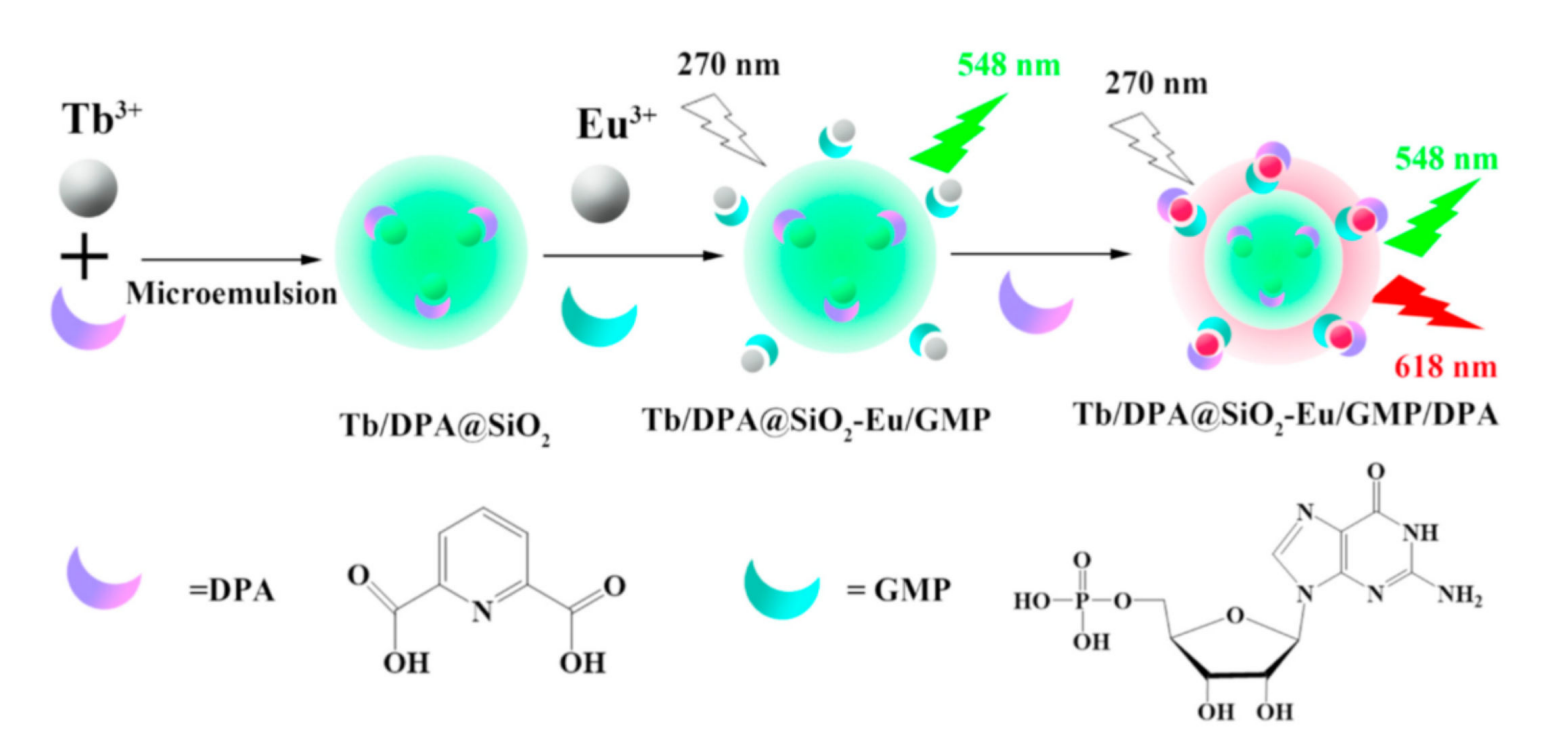
Schematic representation of Tb^3+^/DPA@SiO_2_-Eu^3+^/GMP (Guanosine 5′-monophosphate disodium salt) fluorescent probe formation. DPA, dipicolinic acid. Reproduced from Ref. [91] with permission from the Elsevier.

Transparent, non-toxic and hydrophilic silica nanoparticles (SNs) are of greater impact on sensing and imaging among other polymeric nanoparticles [85,86]. However, conditions of alkaline- or acid-catalyzed hydrolysis of tetraethoxysilane are unfavorable for safe doping of 1,3-diketonate lanthanide complexes into silica matrix due to hydrolysis of lanthanide ions [28,81]. The aforesaid problem can be overcome by enhancing of the thermodynamic stability of the lanthanide complexes [87,88,89,90]. In the case of the tris-1,3-diketonates the stability of their lanthanide complexes can be greatly enhanced by means of the ternary complex formation with N-heterocyclic compounds or phosphine oxides. It is worth discussing the work of Wang group [91], since it demonstrates the safe inclusion of the Tb^3+^ complexes with DPA (shown in Figure 9) into silica seeds with further silica coating through the alkaline-catalyzed hydrolysis of tetraethoxysilane (TEOS). The synthetic scheme in Figure 9 also shows the embedding of Eu^3+^ complexes onto silica surface as the prerequisite for the sensing function of the Tb-Eu-silica nanoparticles, which, in turn, derives from the substrate-induced ternary complex formation. The doping of Eu^3+^ complexes in the form of the heterometallic Eu–Ir nanostructures is also the route for increasing the complex stability [92].

**Figure 10:**
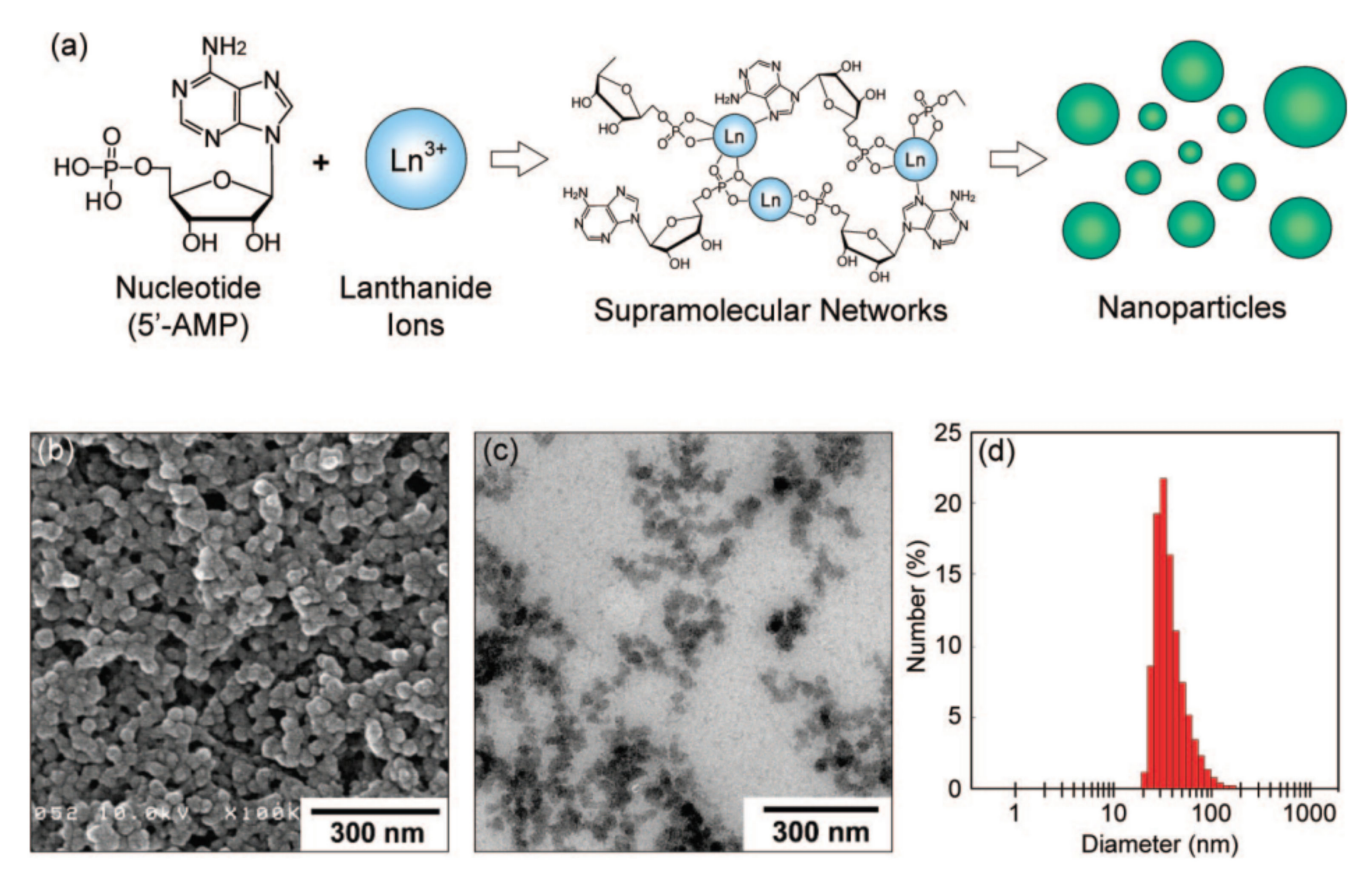
(**a**) A schematic illustration of nanoparticle formation through the self-assembly of 5′-AMP (5′-adenosinemonophosphate) and Gd^3+^ ions. (**b**) SEM image of 5′-AMP/Gd^3+^ nanoparticles prepared in water. (**c**) TEM image of 5′-AMP/Gd^3+^ nanoparticles dispersed in aqueous poly(sodium 4-styrene sulfonate). (**d**) Size distribution histogram obtained by dynamic light scattering (DLS) measurement. Reproduced from Ref. [97] with permission from the American Chemical Society.

The covalent functionalization of 1,3-diketonate lanthanide complexes onto silica surface is also well documented as the route for their embedding onto SNs [93,94]. However, search for facile and easy synthetic route for safe conversion of 1,3-diketonate lanthanide complexes into nanoparticulate form is still challenging task. The nanoprecipitation route is widely applied for transformation of different lanthanide complexes into nanoparticles [95,96]. This method can be based on generation of the nano-aggregates from water soluble molecules and ions as it is shown in Figure 10 [97].

**Figure 11:**
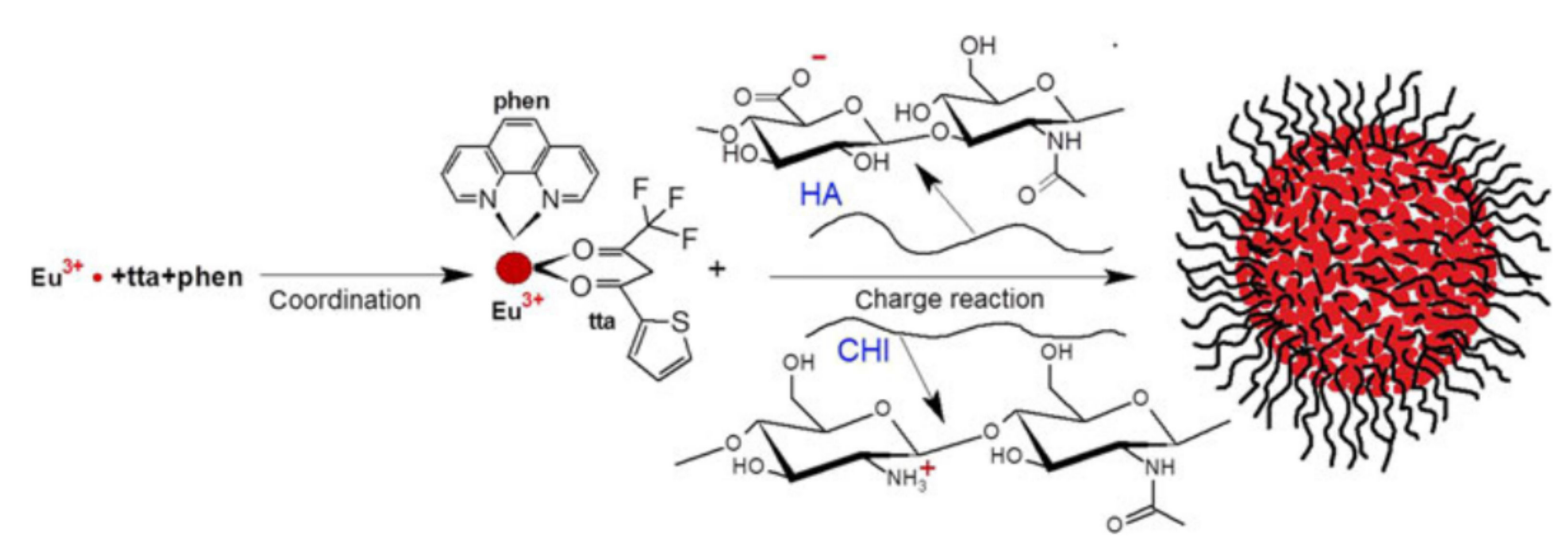
Formation of polyelectrolyte complexes containing Eu^3+^-tta-phen (phenantroline) and stabilized by hyaluronic acid and chitosan. Reproduced from Ref. [98] with permission from the Springer Nature.

The nanoprecipitation of the water insoluble lanthanide complexes with 1,3-diketonates can be performed through the modified solvent-exchange procedure [98]. The procedure is based on both nanoprecipitation of the complexes in the aqueous solutions of polyelectrolytes and adsorption of the latter at the nanoprecipitates (Figure 11). The adsorption of polyelectrolytes forms an exterior layer of the nanoprecipitates, which, in turn, provides their colloid stabilization. The latter was applied for transformation of Eu^3+^ complexes (exemplified by TTA^–^, which is thenoyltrifluoroacetonate) into water dispersible nanoparticulate form [81,99,100,101]. However, the coated by polystyrenesulfonate (PSS) Eu(TTA)_3_-based cores suffers from instability under the pH values above 8.0 or below 6.0. The stability of the Eu(TTA)_3_-based colloids can be greatly increased by means of the ternary complex formation with phosphine oxide derivatives (PhO) [101,102]. The procedure provides very good alternate to other synthetic routes for doping or deposition of lanthanide complexes into or onto silica nanoparticles. The advantage of the polyelectrolyte-coated morphology is the layer-by-layer polyelectrolyte deposition driven by an electrostatic attraction. In particular, the positively charged nanoprecipitates favor the adsorption of negatively charged PSS, while the negatively charged PSS exterior layer is the prerequisite for the adsorption of polyethyleneimine (PEI), which can be followed by the PSS-layer [101].

The presence of anionic groups in the exterior polyelectrolyte layer also provides the route for inclusion of the lanthanide ions. The incorporation of Ln^3+^ ions into the polyelectrolyte layer has been exemplified by either coordination of Tb^3+^ ions with carboxylates of polyacrylic acid [103] or encapsulation of Eu^3+^ 1,3-diketonate complex into the polyelectrolyte layer through the charge-dipole interactions [104,105].

**Figure 12:**
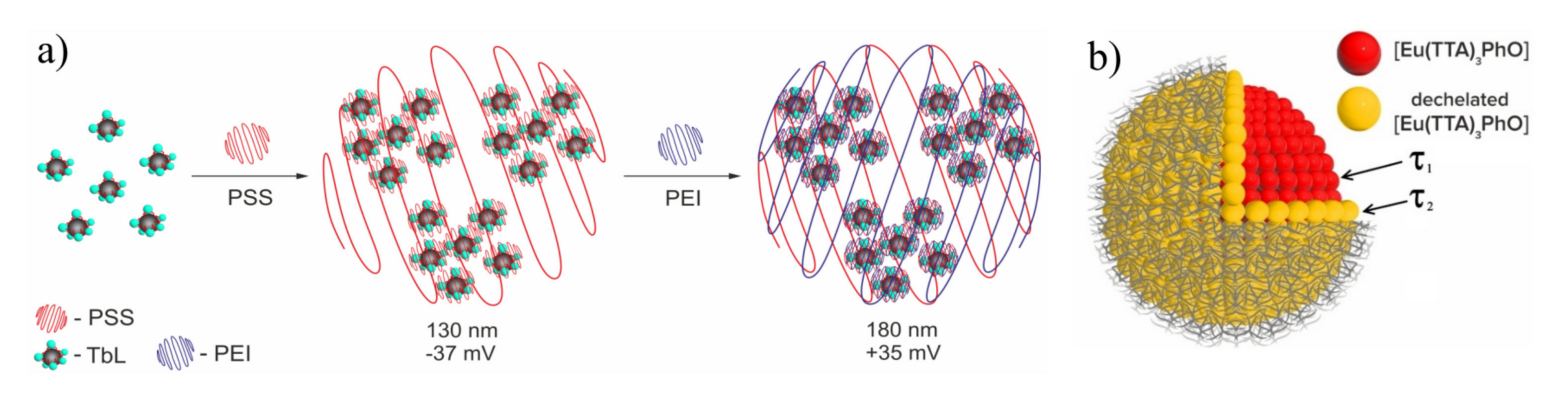
(**a**) Schematic illustration of nanoprecipitation method of nanoparticles formation. (**b**) Schematic illustration of two types of lanthanide-based blocks with different surface and bulk [Eu(TTA)_3_PhO] localization. Reproduced from Refs. [106,107] with permission from the Elsevier.

As it has been mentioned above the embedding of 1,3-diketone moieties onto the cyclophanic backbone both favors the bis-chelation of lanthanides and facilitates the nanoprecipitation of the lanthanide complexes in the framework of the solvent-exchange-based synthetic route. This enables to convert the lanthanide complexes with the cyclophanic derivatives of 1,3-diketonates into water dispersible nanoparticles through the modified nanoprecipitation procedure as it is shown in Figure 12a.

The nanoprecipitation of the lanthanide complexes results in two types of lanthanide-based building blocks with different inner- and outer-sphere environment, as it is schematically illustrated in Figure 12b. These two types of the lanthanide complexes are manifested by the two-exponential decay which, in turn, can be ascribed to the presence of different environments around the complexes. The latter may depend on position of the luminescent complexes inside or at the surface of the nanoparticles, where the presence of local defects and interface states may provide non-radiative recombination paths [106,108]. The specific bis-chelating coordination mode of lanthanide ions in the complexes with cyclophanic 1,3-diketonates provides greater stability than the mono-chelation of TTA^–^. This is manifested by the invariance of the lanthanide-centered luminescence of the PSS-coated nanoprecipitated terbium complexes with the cyclophanic 1,3-diketone derivatives which will be further designated as [TbL] (L is cyclophanic 1,3-diketonate) at the pH values varied within wide pH-range (4-9) [53]. The easy manipulation with the PSS-stabilized colloids through the centrifugation-induced phase separation and further re-dispersion in different solutions enables their re-usability, which, in turn, facilitates their applicability in sensing [53,61,101,106,107,108,109,110,111,112,113,114,115].

**Figure 13:**
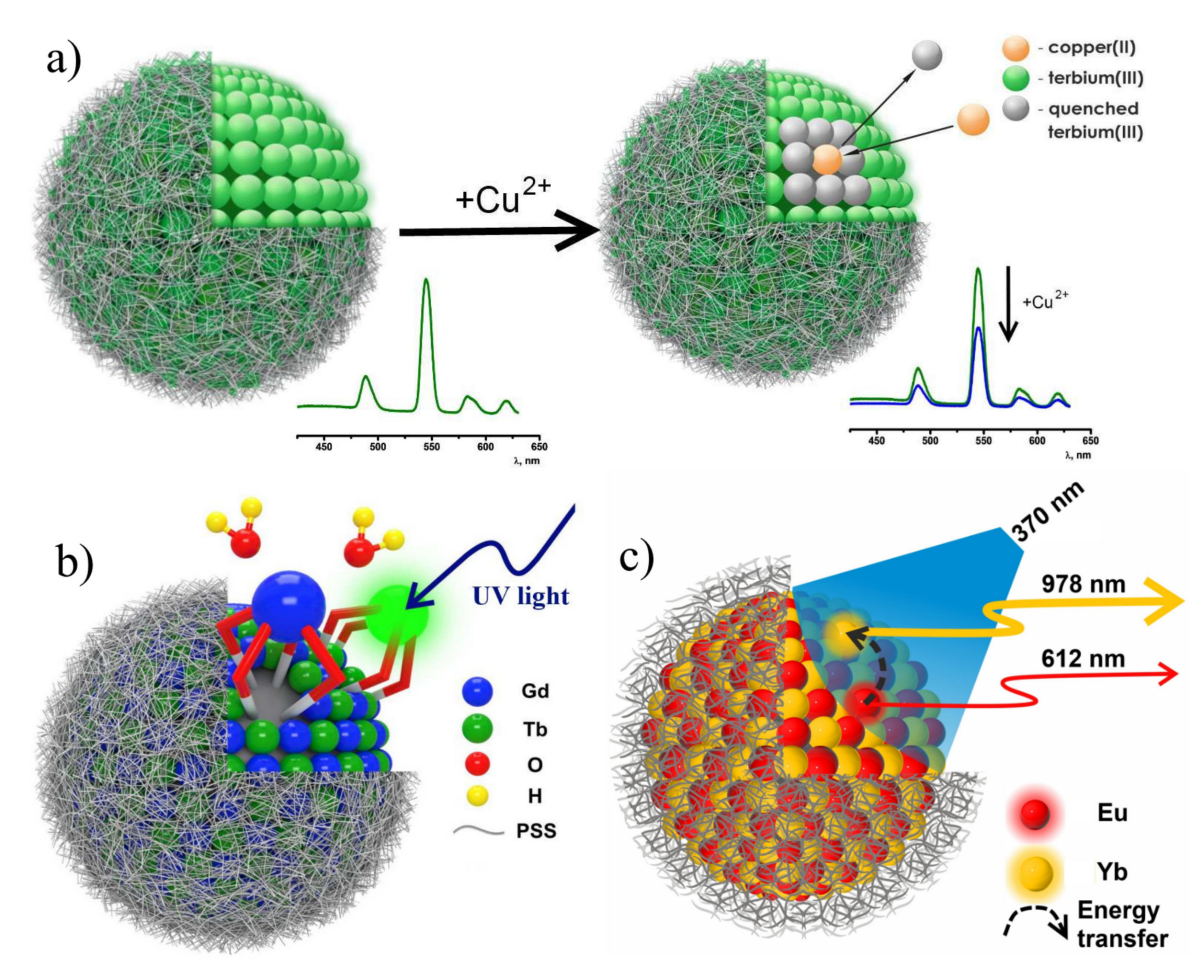
(**a**) Illustrative demonstration of Tb^3+^-centered luminescence quenching by copper(II) ions. (**b**) Schematic structure of heterometallic Tb^3+^ and Gd^3+^ PSS-coated nanoparticles. (**c**) Representation of Eu→Yb energy transfer in PSS-[Ln (TTA)_3_ PhO] colloids. Reproduced from Refs. [61,108,106] with permission from the Springer Nature and the Elsevier.

The high activity and coordinative unsaturation of the lanthanide centers located at the interfacial layer is the prerequisite for the sensing of antibiotics of fluoroquinolone and tetracycline series, where the ternary complex formation triggers the pronounced answer of the terbium-centered luminescence [100,109]. The embedding of the specific N-heterocyclic moieties onto lower rim of calix[4]arene bearing 1,3-diketone moieties at the upper rim (**41f**) provides the binding sites for both terbium and copper ions [61]. In turn, the Cu^2+^–Tb^3+^ heterometallic complex formation should be followed by the quenching of the Tb^3+^-centered luminescence (Figure 13a). The quenching effect is the basis for sensing of copper ions at very low concentration level. The quenching effect is contributed by both steady state and dynamic quenching mechanisms, in turn, derived from the ions exchange and donor-acceptor energy transfer correspondingly [61]. Moreover, the nature of the exterior layer exemplified by PEI provides the sensing of copper ions through the luminescence of [Eu(TTA)_3_PhO]-based colloids (PhO is the phosphine oxide derivative) [81]. The separation of copper and terbium ions due to their predominant localization in the exterior and interior zones of the Eu(TTA)_3_-based colloids coated by PSS-PEI bilayer is the prerequisite for the great contribution of the dynamic quenching mechanism (Figure 13a).

The close similarity in the coordination properties of the neighboring lanthanide ions, exemplified by Tb^3+^ and Gd^3+^ makes their complexes with 1,3-diketone calix[4]arenes structurally similar (Figure 13b), which enables to evaluate the triplet level of the ligand from the ligand-centered luminescence of Gd^3+^ complexes with the cyclophanic 1,3-diketone (**27a**). The structural similarity also favors a combination of the Tb^3+^ and Gd^3+^ complexes within each nanoprecipitate when the complexes are mixed the DMF solution with further nanoprecipitation in the aqueous-DMF solution of PSS [108]. The combination of the Tb^3+^- and Gd^3+^-counterparts enables to reveal the concentration-induced quenching of the Tb^3+^-centered luminescence. The similar effect was detected for Eu(TTA)_3_PhO complexes converted into the PSS-coated colloids under their dilution by the Lu^3+^- and Yb^3+^-counterparts [106]. The difference in photophysical properties of homometallic and heterometallic colloids reveals the energy transfer between the lanthanide complexes constituting the nanoprecipitates. The latter is either restricted or enhanced under their self-assembly within heterometallic nanoparticles which, in turn, can be the reason for tuning their photo-physical properties. In particular, the concentration induced quenching in the homometallic nanoparticles with Eu^3+^-centered luminescence can be significantly decreased in the Eu^3+^-Lu^3+^-based colloids derived from the self-assembly of luminescent Eu^3+^ and non-luminescent Lu^3+^ isostructural complexes. The quenching of Eu^3+^-centered luminescence in the Eu^3+^-Yb^3+^ nanoparticles due to Eu→Yb energy transfer is accompanied by increase in the NIR luminescence of Yb^3+^ complexes (Figure 13c).

**Figure 14:**
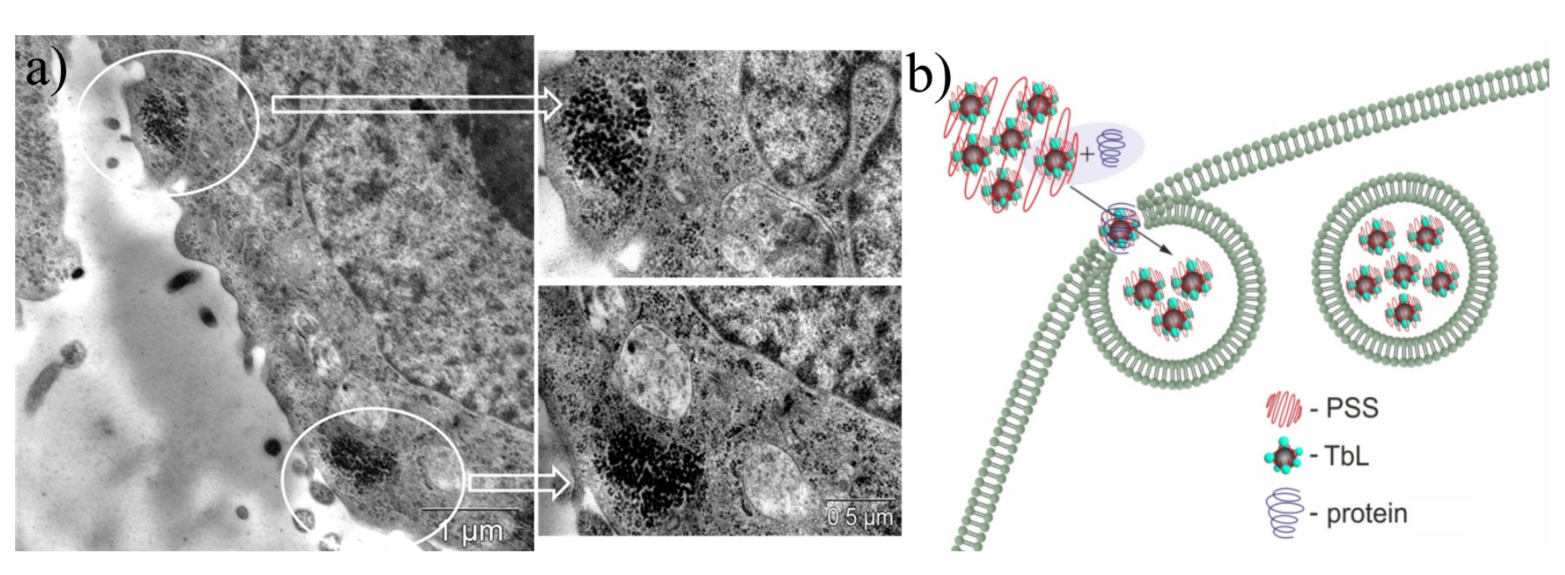
(**a**) TEM image of Hep-2 (human larynx carcinoma) cells incubated with PSS-[Tb**28a**] colloids. White circles designate the localization of the nanoparticles. (**b**) Schematic illustration of BSA (bovine serum albumin) assisted cell internalization of PSS-[Tb**28a**] colloids. Reproduced from Ref. [107] with permission from the Elsevier.

The conversions of the Tb^3+^ complexes with cyclophanic 1,3-diketonates into the PSS-based colloids makes them promising basis for the cellular imaging. The staining of the cell cytoplasm by the green luminescence of terbium complexes with 1,3-diketone calix[4]arene (**28a**) results from the cell internalization of their PSS-coated colloids [107]. It is worth noting that the negative charge of the PSS-based exterior layer of the colloids doesn’t prevent their cell internalization, while the double-layered PEI-PSS-coated colloids with positively charged exterior layer exhibit worse cell internalization versus the PSS-coated colloids (Figure 14) [107]. This tendency is explained by the enhanced aggregation of the double layered colloids versus the mono layered ones. In turn, the cellular uptake behavior of the PSS-coated colloids in great extent derives from the so-called protein corona arisen from the interactions with the blood serum proteins in the cell samples (Figure 14a,b). The use of the [Eu(TTA)_3_PhO]-based colloids enables to stain the cell cytoplasm by red Eu-centered luminescence [106].

**Figure 15:**
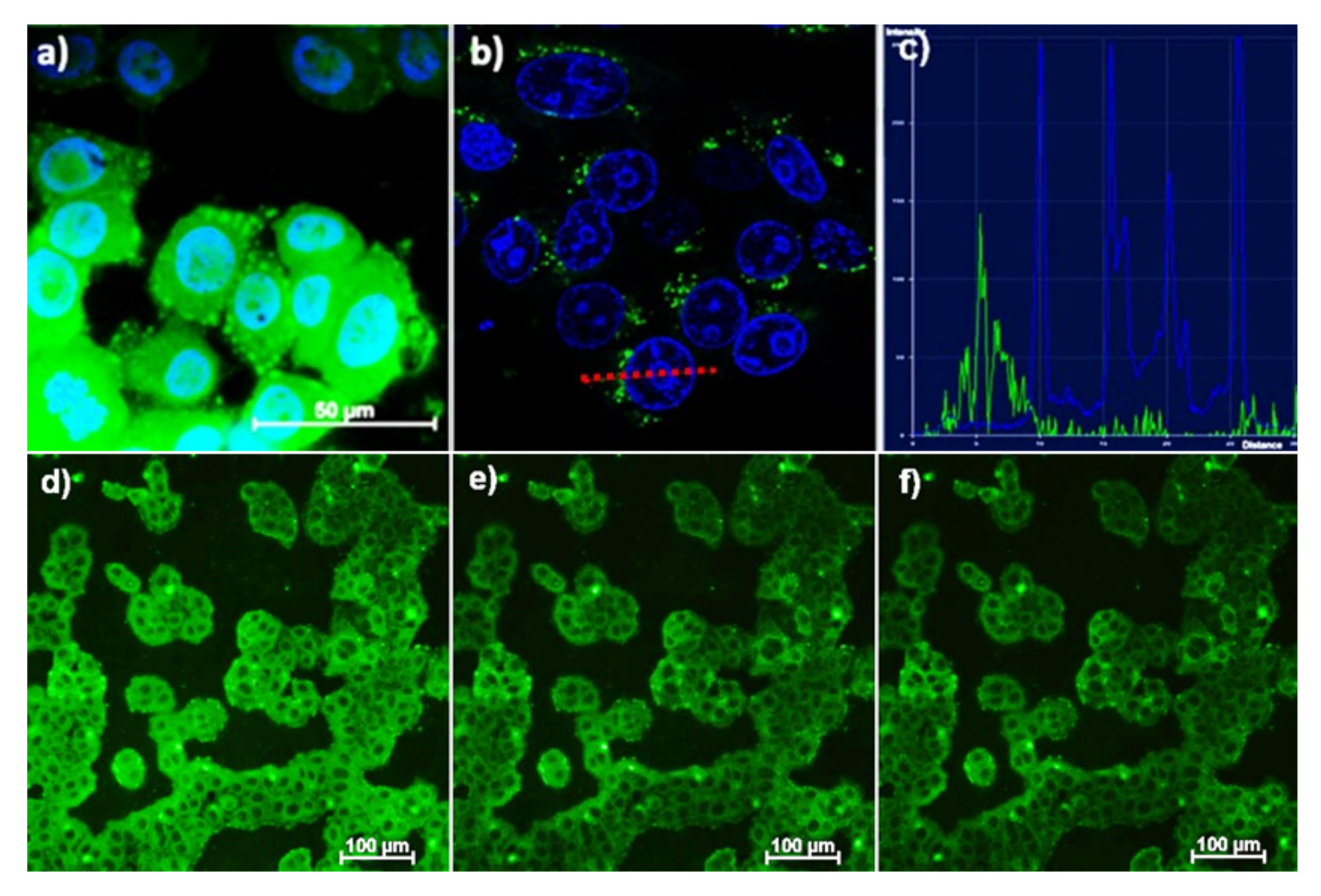
Fluorescent microscopy (**a**) and confocal microscopy (**b**) images of M-Hela cells incubated with PSS-[Tb_2_(**43**)_2_]. The cell nuclei are stained by DAPI (**a**) and Hoechst 33342 (**b**). The graph of luminescence intensity of Hoechst 33342 (blue) and nanoparticles (green) (**c**) between two points, designated on (**b**) with red line, demonstrating the nanoparticles location outside the cell’s nucleus. Fluorescent microscopy images of M-Hela stained by PSS-[Tb_2_(**34**)_2_] at different temperatures (**d**) 30 °C, (**e**) 35 °C, (**f**) 40 °C [114].

Our work [111] is worth mentioning as an example of sensing ability of the PSS-coated colloids based on the terbium dinuclear complexes with two thiacalix[4]arene ligands ([Tb_2_(L)_2_]) (L = **34**, **43**). As it has been mentioned above, the energy of the ligand’s triplet energy in [Tb_2_(L)_2_] (L = **34**, **43**) complexes facilitates the temperature-induced quenching resulted from the enhancement of the back energy transfer. The transformation of the dimeric complexes into the PSS-coated colloids prerequisites their intracellular localization, which, in turn, provides the applicability of PSS-[Tb_2_(L)_2_] (L = **34**, **43**) nanoparticles as highly sensitive intracellular temperature nanosensors (Figure 15a–f).

The transmission electronic microscopy (TEM) analysis reveals that the average size of the nanoprecipitates is at the level of 4–12 nm [53,107,109,113]. It is worth noting that such size level is favorable for high extent of the Ln^3+^ centers located at the water/nanoparticle interface [116]. The interfacial localization of the metal centers in the colloids facilitates their accessibility to water molecules from the bulk of solution. This fact along with the high hydration number of the Ln^3+^ complexes with 1,3-diketone calix[4]arenes is the reason for high longitudinal relaxivities of the colloids based on the Gd^3+^ complexes with calix[4]arene 1,3-diketonates [108,110]. This, in turn, is the prerequisite for efficient contrasting effect in NMR imaging [111]. The longitudinal and transverse relaxivity values (r_1_ and r_2_) measured in the aqueous colloids of the PSS-coated colloids based on the Gd^3+^ complexes with the cyclophanic 1,3-diketones are within 14-20 mM^−1^∙s^−1^ (**26**, **27a**) [108,110]. These values are higher than those reported for the Gd^3+^-based commercial contrast agents [116] due to the slowed down rotational and translational motion of the Gd-centers after their transformation from molecular to nanoparticulate form.

**Figure 16:**
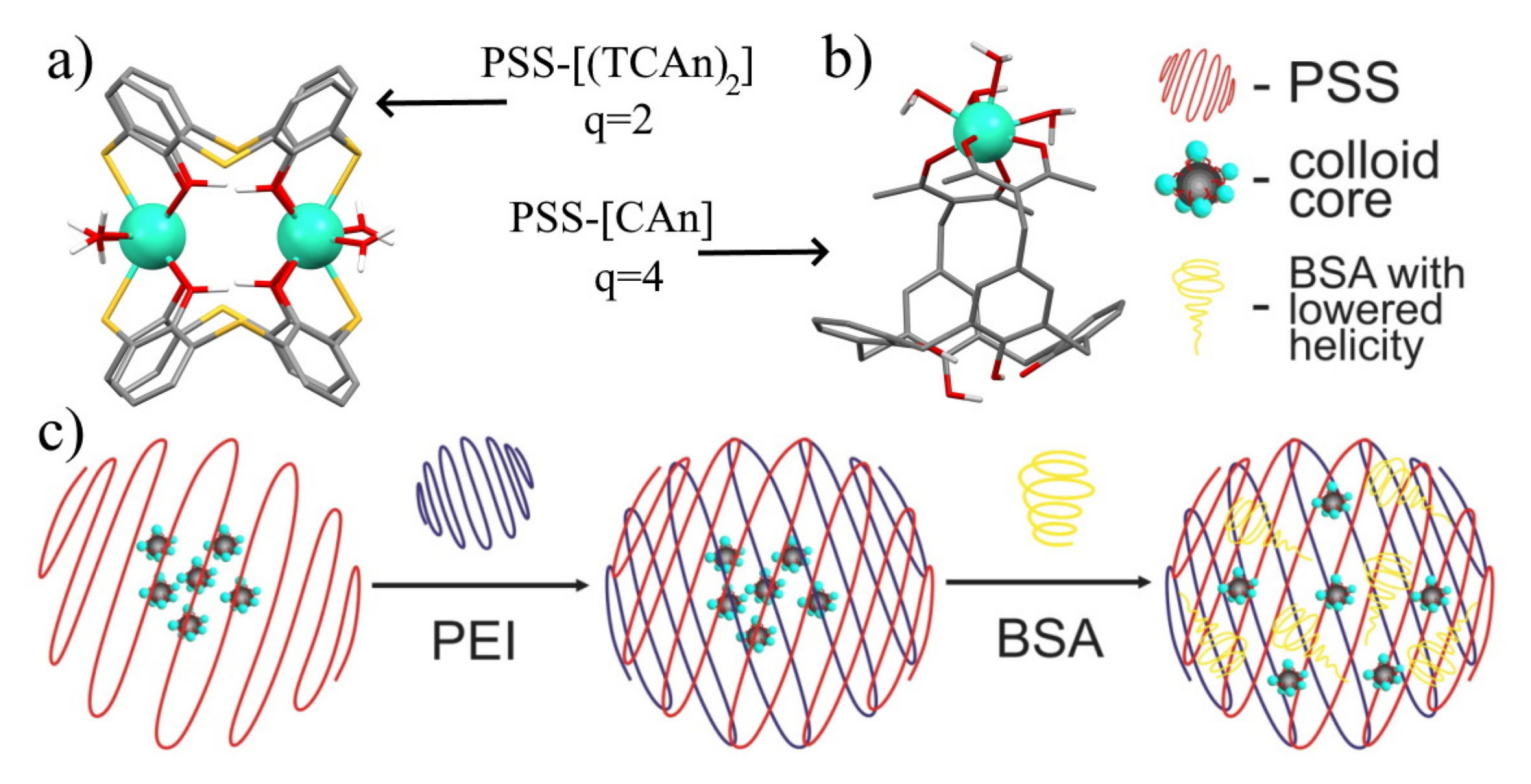
Schematic presentation of the coordination modes of Ln^3+^ ions in (**a**) [Ln_2_(TCAi)_2_] (TCAi = **34, 42**) and (**b**) [LnCAi] (Cai = **32a**, **32b**, **37**) complexes and their corresponding hydration numbers, q. (**c**) Schematic presentation of PSS-Na[Gd_2_**26**], PEI-PSS-Na[Gd_2_**26**], and BSA-PEI-PSS-Na[Gd_2_**26**] colloids. Reproduced from Refs. [109,113] with permission from the Elsevier.

As it has been mentioned above, the Gd^3+^ complexes with the cyclophanic 1,3-diketone derivatives are isostructural with the terbium ones, which facilitates the joint study of both Tb^3+^- and Gd^3+^ counterparts. The latter, in turn, enables to evaluate the hydration number of the Ln^3+^ centers in the colloids by measuring of the time resolved Tb^3+^-luminescence in H_2_O and D_2_O solutions for highlighting an impact of the hydration number on the relaxivity values of the colloids [111,114]. The joint study of both Tb^3+^- and Gd^3+^ counterparts of the PSS-coated colloids based on calix[4]arene 1,3-diketones (**32a**, **32b**, **37**) and thiacalix[4]arenes (**34**, **42**) (Figure 16a,b) enabled to highlight the impact of the coordination modes of Gd^3+^ ions by the aforesaid ligands on both Tb^3+^-luminescence and Gd^3+^-induced paramagnetic enhancement of the water protons [111,114]. The low hydration number derived from the binuclear sandwich-like coordination of Ln^3+^ ions by two thiacalix[4]arenes (**34**, **42**) (Figure 16a) facilitates the Tb^3+^-luminescence of their colloids in comparison with those based on Tb^3+^ complexes with the cyclophanic 1,3-diketonates (**32a**, **32b**, **37**) (Figure 16b). However, the r_1_ and r_2_ values of the colloids based on Gd^3+^-counterparts with thiacalix[4]arene are lower (1.8–2.6 and 2.7–3.9 mM^−1^s^−1^) than those measured for complexes with calix[4]arene 1,3-diketonates with the greater hydration number [111]. Moreover, the joint study of the isostructural Tb^3+^ and Gd^3+^complexes with calix[4]resorcinarene 1,3-diketone as building blocks of the PSS-coated colloids facilitated to reveal the effect of their aggregation on r_1_ and r_2_ values [113]. In particular, the enhanced aggregation derived from the double layered (PEI-PSS) coating of the nanoprecipitates restricts the accessibility of the Gd^3+^-centers to water molecules, which is manifested by the decrease in the r_1_ and r_2_ values. In turn, these values tend to increase under the de-aggregation of the colloids induced by their interaction with the blood serum proteins (Figure 16c) [113].

**Figure 17:**
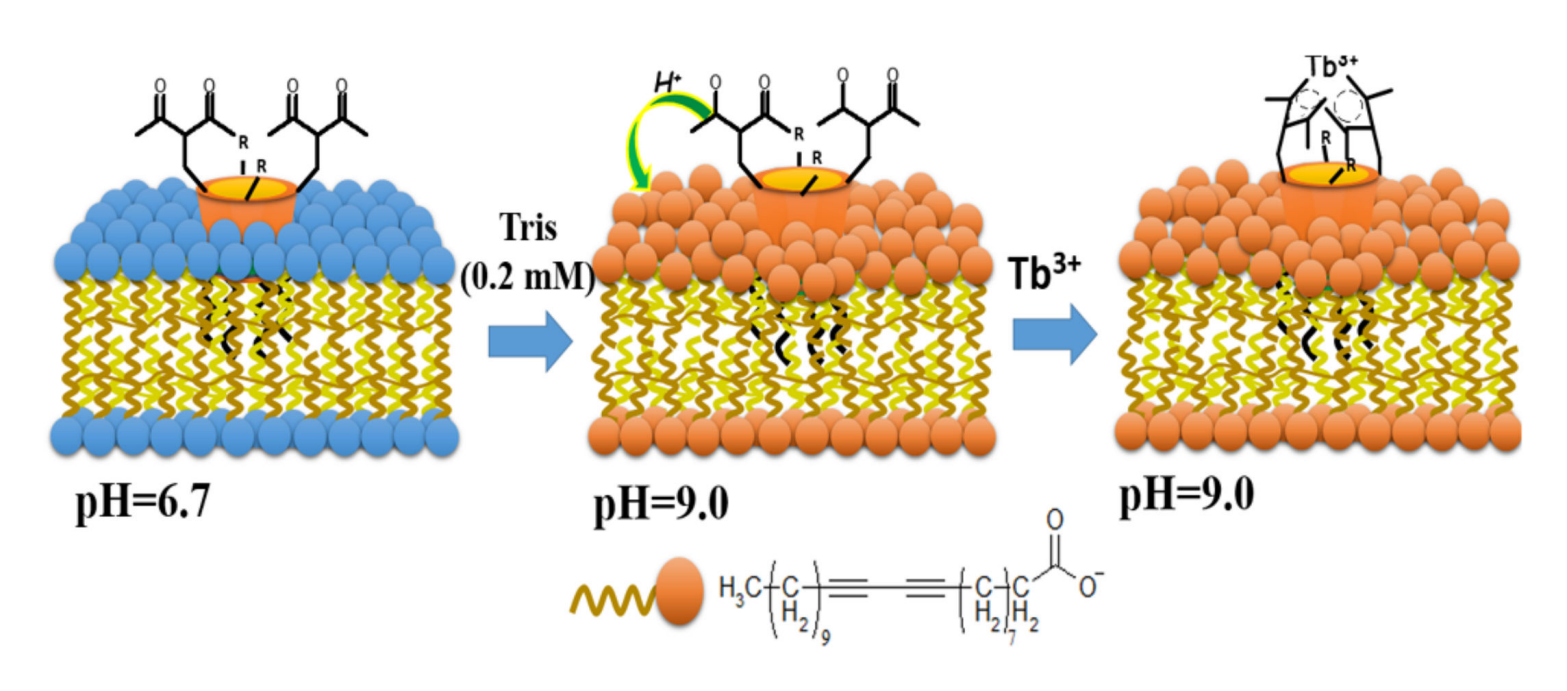
Schematic illustration of proton-pump effect of the carboxylate-groups of polydiacetylene (PDA)-vesicles on the coordination of Tb^3+^ ions via 1,3-diketonate groups of **28b** and **41d** embedded into the PDA vesicles. Reproduced from Ref. [117] with permission from the Elsevier.

The great advantage of the calix[4]arene backbone is the ability to embed the functional groups for controlling the hydrophobicity or hydrophilicity of both lower and upper rims of calix[4]arene. The increased hydrophobicity of the calix[4]arene 1,3-diketones can be achieved through their lower rim substitution by nonyloxy-groups (**28b**, **41d**). The latter enables to incorporate the nonyl-substituted calix[4]arene 1,3-diketones into polydiacetylene (PDA) vesicles resulting in the formation of the mixed water dispersible aggregates, where the nonyl groups are included into the hydrophobic layer of PDA vesicles, while hydrophilic 1,3-diketone moieties are exposed at the hydrophilic surface. The nano-architecture of the mixed vesicles is schematically illustrated in Figure 17. The proximity of 1,3-diketone to carboxy/carboxylate groups facilitates the diketone-to-1,3-diketonate transformation resulting in coordination of Tb^3+^ ions which, in turn, is followed by the enhancement of the Tb^3+^-centered luminescence [117].

## 7. Conclusions

The above presented results highlight synthetic routes for achieving the structural diversity of cyclophanic 1,3-diketones as versatile ligands for the formation of lanthanide complexes exhibiting both lanthanide-centered luminescence and magnetic relaxivity parameters convenient for contrast effect in MRI. The relationship between structural features of the cyclophanic 1,3-diketones and their complex ability is considered as a tool to control both functional properties of the produced complexes and their conversion into nanoparticulate forms. It is worth noting the advantages of the 1,3-diketone calix[4]arene-based ligands in developing of sensors and contrast agents.

(1) The embedding of two or four 1,3-diketone moieties onto the cyclophanic backbone favors the bis-chelating coordination mode of lanthanide ions. The tight binding of the bis-chelated lanthanide ions along with the low water solubility of the cyclophanic ligands facilitates safe conversion of the lanthanide complexes into the water dispersible nanoparticulate form.
(2) The bis-chelating coordination mode of the lanthanide ions both remains enough hydration numbers in the lanthanide complexes with 1,3-diketone calix[4]arenes and restricts leaching of Ln^3+^ ions from the nanoprecipitates. This is the prerequisite for their sensing ability in aqueous media and efficient contrast effect in MRI.
(3) The presence of the calix[4]arene backbone opens the opportunity of embedding different substituents onto lower or upper cyclophanic rims as a tool for tuning the terbium- or ytterbium-centered luminescence. Moreover, it is also shown that the embedding of the hydrophobic substituents enables to diversify the nanoparticulate forms of the Tb^3+^ complexes.
(4) The wide range of topologies of the cyclophanic backbones—calix[4]arene, calix[4]resorcinarene or thiacalix[4]arene—provides another tool for tuning both magnetic relaxation and luminescent properties through the structural diversity of the coordination modes of lanthanide ions. In turn, the substitution of the thiacalix[4]arene ligand enables to control its antenna effect on the terbium-centered luminescence through the modification of the triplet energy level.

Finally, from the results presented above it is clear that the cyclophanic 1,3-diketones can be considered as versatile building blocks for the construction of new nanosensors, fluorescent cellular contrast agents and gadolinium-containing contrast agents for MRI with improved functional parameters. We hope that this review will both open a new chapter of molecular design of 1,3-diketone calix[4]arenes and facilitate their use in various applications.

## Figures and Tables

**Table 1:** The content (%) of enol tautomer for calix[4]arenes in CDCl_3_ (0.03M), energies of lowest triplet states (*T*_1_) of the ligands in the Tb^3+^ complexes, average lifetimes of the Tb^3+^-centered luminescence (<τ>^a^) and ligand-centered phosphorescence of the Gd^3+^ complexes (<τ>^b^), as well as relative thermal sensitivity (*S_I_*) values at various temperatures in DMF.

L		R_1_	R_2_	R_3_	R_4_	R_5_	Enol Form, %	*T*_1_, λ, nm (ν, cm^-1^) at 146 K	<τ>^a^ (μs) at 298 K (323K)	<τ>^b^ (μs) at 146 K	*S_I_* at 293 K (323K)	References
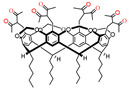	**26**						~5-10					[53]
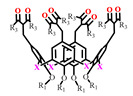	**X** = CH_2_											
	**27a**	H	-	CH_3_	-	-	40	457 (21882)	20	633	-	[54,58,69]
	**27b**	H	-	*t*-Bu	-	-	<0.2		494	-	-	[58]
	**28a**	C_3_H_7_	-	CH_3_	-	-	50	-	150	-	-	[54]
	**28b**	C_9_H_19_	-	CH_3_	-	-	33	442 (22624)	98	558		[70]
	**X** = S											
	**29a**	H	H	CH_3_	-	-	40	444 (22522)	358	971	-	[58,69]
	**29b**	H	H	*t*-Bu	-	-	<0.2	-	544	-	-	[58]
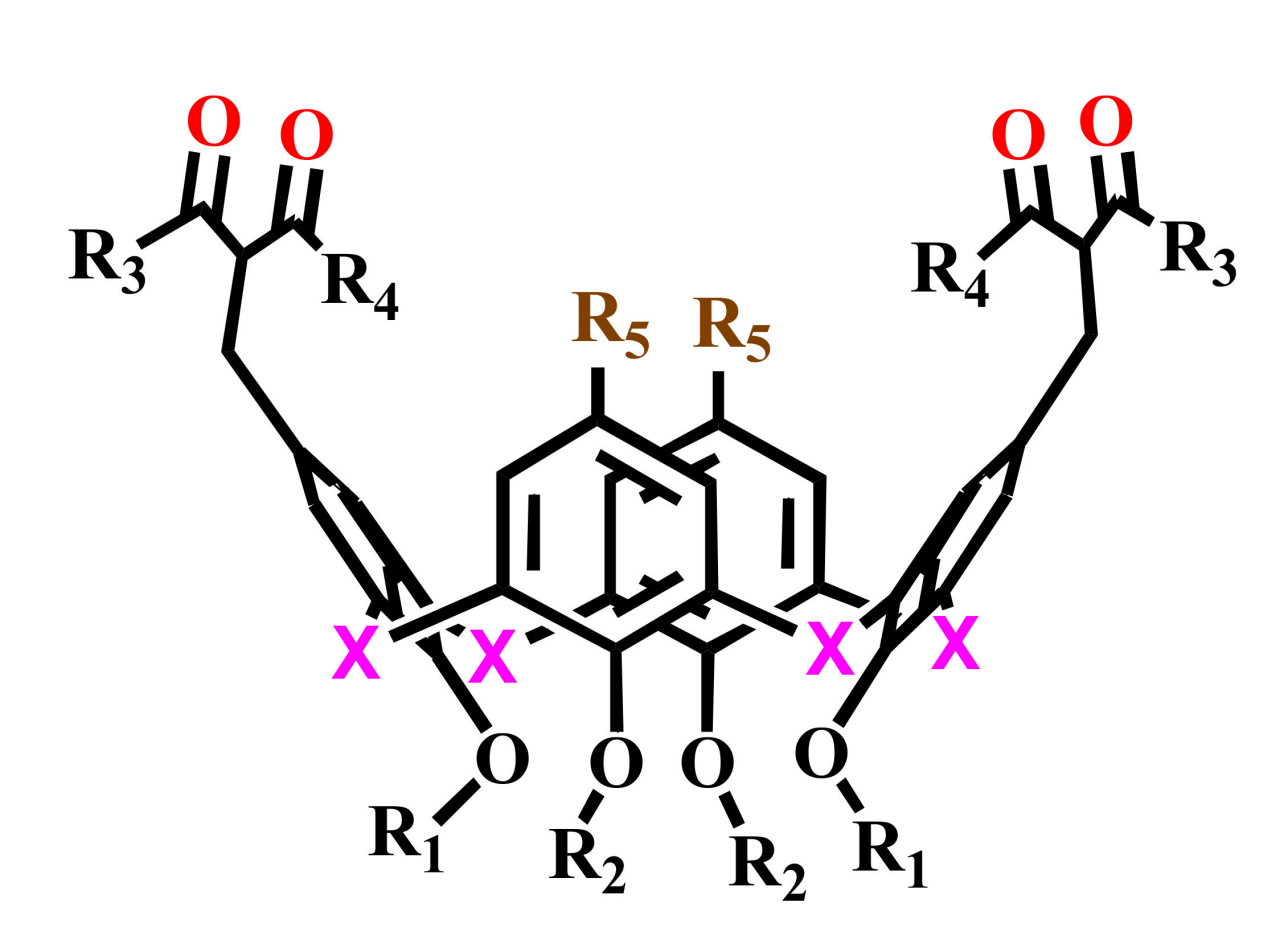	**X** =CH_2_											
	**32a**	H	H	CH_3_	CH_3_	H	39	441 (22670)	127	738	-	[55,59]
	**32b**	H	H	CH_3_	Ph	H	<0.1	-	-	-	-	[55]
	**32c**	H	H	Ph	Ph	H	<0.1	-	-	-	-	[55]
	**37**	H	H	CH_3_	CH_3_	Br	30	438 (22830)	200	927	-	[59]
	**41a**	C_3_H_7_	C_3_H_7_	CH_3_	CH_3_	Br	35	430 (23250)	220	785	-	[59]
	**41b**	C_3_H_7_	C_3_H_7_	CH_3_	Ph	Br	8	470 (21270)	-	1023	-	[59]
	**41c**	C_3_H_7_	C_3_H_7_	Ph	Ph	Br	~0	-	-	-	-	[59]
	**41d**	C_9_H_19_	C_9_H_19_	CH_3_	CH_3_	Br	38	429(23310)	114	865	-	[70]
	**41e**	C_3_H_7_	2-CH_2_Py	CH_3_	CH_3_	Br	41	433 (23095)	136	963	-	[61]
	**41f**	C_3_H_7_	2-CH_2_Quin	CH_3_	CH_3_	Br	45	452 (22124)	141	611	-	[61]
	**X** = S											
	**38**	H	H	CH_3_	CH_3_	Br	42	452 (22124)	1030 (940)	785	1.84 (3.79)	[60]
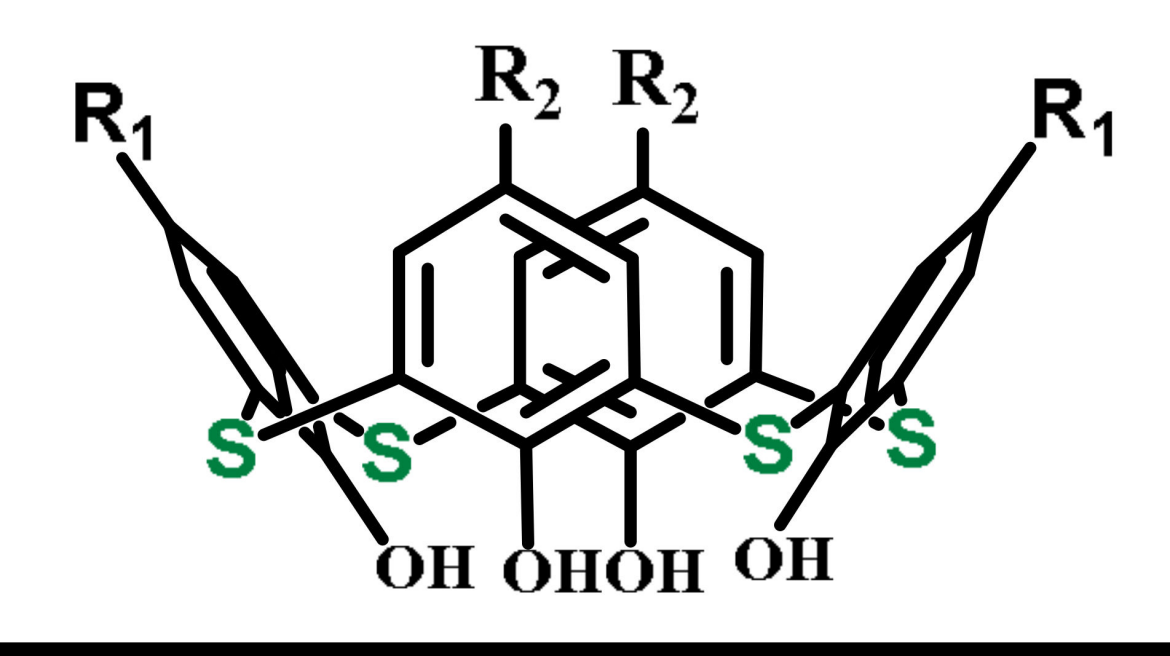	**34**	H	Br					440 (22727)	1120 (990)	421	1.67 (3.99)	[60]
	**42**	H	H				-	417 (23981)	1090	1437	0.156 (0.165)	[60,69]
	**43**	Br	Br					458 (21834)	980 (842)	301	2.06 (5.74)	[60]

## Data Availability

Not applicable.
